# Enhancing upper limb motor recovery prediction after acute stroke using EEG and subacute data

**DOI:** 10.1063/5.0287165

**Published:** 2026-02-02

**Authors:** Michael Lassi, Stefania Dalise, Luigi Privitera, Nicola Giannini, Michelangelo Mancuso, Valentina Azzollini, Tommaso Ciapetti, Antonello Grippo, Silvestro Micera, Francesca Cecchi, Alberto Mazzoni, Carmelo Chisari, Andrea Bandini

**Affiliations:** 1 The Biorobotics Institute and Department of Excellence in Robotics and AI, Scuola Superiore Sant'Anna, Pisa, Italy; 2 Neurorehabilitation Unit, University Hospital of Pisa, Pisa, Italy; 3 School of Advanced Studies, University of Camerino, Camerino, Italy; 4 Unit of Neurology, University Hospital of Pisa, Pisa, Italy; 5 Department of Clinical and Experimental Medicine, Neurological Institute, University of Pisa, Pisa, Italy; 6 Department of Translational Research and New Technologies in Medicine and Surgery, University of Pisa, Pisa, Italy; 7 IRCCS Fondazione Don Carlo Gnocchi, Florence, Italy; 8 UO Neurofisiopatologia, Azienda Ospedaliera Universitaria Careggi, Florence, Italy; 9 Translational Neural Engineering Laboratory, Neuro-X Institute, École Polytechnique Fédérale de Lausanne (EPFL), Lausanne, Switzerland; 10 Modular Implantable Neuroprostheses (MINE) Laboratory, Università Vita-Salute San Raffaele, Milan, Italy; 11 Department of Experimental and Clinical Medicine, University of Florence, Florence, Italy; 12 Health Science Interdisciplinary Research Center, Scuola Superiore Sant'Anna, 56127 Pisa, Italy

## Abstract

Electroencephalography (EEG) has shown promise in assessing and monitoring functional recovery in stroke survivors, but its utility in predicting upper limb motor recovery in a data-driven framework remains underexplored. This study presents a novel EEG-based machine-learning model, *StrokeRecovNet*, developed to predict motor recovery outcomes based on the upper extremity subscale of the Fugl-Meyer Assessment (FMAUE). *StrokeRecovNet* is a feed-forward neural network optimized for regression tasks, leveraging 221 candidate EEG biomarkers, spanning spectral and functional connectivity domains, along with baseline clinical information. These inputs are used to predict follow-up FMAUE scores in stroke survivors who underwent standard rehabilitative protocols. We validated our pipeline on two independent datasets of patients in the acute and subacute post-stroke phases. *StrokeRecovNet* consistently outperformed the proportional recovery rule (PRR), a standard benchmark based on initial impairment, in predicting FMAUE scores in the subacute stage (median absolute error, MAE: *StrokeRecovNet* = 5.85, PRR = 19.00). Incorporating support data from the subacute dataset led to improved predictive performance in the acute sample (MAE: *StrokeRecovNet* = 5.87, PRR = 8.80), whereas the model trained solely on the acute data did not (MAE: 13.74). Key features contributing to the model's success included brain symmetry indices and functional connectivity measures, evolving across recovery stages. These findings demonstrate the potential of EEG-based biomarkers to predict individual recovery trajectories. This work introduces a novel, data-driven approach to forecasting upper limb recovery using EEG and suggests that EEG data from the subacute stage, which is more readily available in clinical settings, can enhance early predictions, paving the way for personalized post-stroke rehabilitation strategies.

## I. INTRODUCTION

Stroke is a major cause of disabilities worldwide, leading yearly to the loss of 143 × 10^6^ disability-adjusted life years.^1^ Although early treatment of stroke impairment may take advantage of a time window of increased brain plasticity,^2^ almost 75% of stroke survivors still suffer from chronic disabilities,^3^ affecting mainly their motor functions.^4^ In this context, upper extremity (UE) impairments are among the most common consequences of stroke, affecting up to 80% of stroke survivors,^5^ with a huge negative impact on people's quality of life and social participation.^6–8^ To optimize the UE rehabilitation process, therapies should consider both the patient's specific needs and the neurological processes behind them. In this direction, tools that can predict patients' ability to recover UE functions are essential to determine the type, duration, and goal of the rehabilitation.^9^

Clinical evidence suggests that the Fugl-Meyer Assessment for the UE (FMAUE)^10^ is a strong predictor of the longitudinal outcome a few months after the stroke onset.^11,12^ This principle follows the so-called *proportional recovery rule* (PRR) that states that throughout the subacute phase after stroke, most patients recover approximately 70% of their total UE function.^13^ The PRR suggests that severe hemiparetic patients typically regain a substantial portion of their lost UE function during the first 3 months post-stroke, largely reflecting the natural recovery trajectory rather than the specific effects of neurorehabilitation. Although the PRR may not be able to identify the entire complexity of recovery patterns, as other statistical models have tried to achieve,^14^ and may be prone to being overly optimistic in the amount of correlation with the initial impairment (due to mathematical coupling^15^), it is still widely used as a starting point to assess plausible recovery after stroke.^16,17^

Nonetheless, there is a subset of patients, comprising approximately 30% of stroke survivors,^11^ characterized by severe hemiparesis whose recovery trajectory deviates from the PRR, referred to as “non-recoverers.” Consequently, these individuals fail to attain the desired UE motor function even after the acute phase.^16,18^ The elusive goal in neurorehabilitation is the identification of predictors capable of discerning non-recoverers within a few hours post-stroke. This quest is considered a main objective of neurorehabilitation, as it holds the potential to unlock personalized treatment strategies and optimize therapeutic outcomes. However, acute stroke patients are often less stable than those in later phases, hence leading to difficulties in acquiring neuroimaging data from them.^19^ This may pose a huge challenge in training machine and deep learning models of prediction, as there is a general lack of acute-phase neuroimaging recordings.

In this context, previous investigations on the neural basis of non-recoverer behaviors have been performed using magnetic resonance imaging (MRI),^20,21^ functional MRI (fMRI),^22^ and transcranial magnetic stimulation.^23^ While effective in identifying markers of recovery, these technologies may not be ideal in the clinical setting because of their cost or setup times. On the contrary, electroencephalography (EEG) provides a cheaper and largely available tool to functionally image the brain condition. EEG has been extensively used in the functional prognosis of stroke survivors.^24–27^ Specifically, the EEG resting-state paradigm is a viable approach for predicting upper-extremity recovery as early as the acute post-stroke stage,^21^ for two main reasons: (1) it is not biased by the patient's ability to perform a specific task, and (2) it can be implemented within the first hours after stroke. Establishing quantitative resting-state EEG markers is therefore essential to both summarize a patient's condition and monitor their upper-extremity recovery over time.

Earlier research focused on the prognostic potential of resting-state EEG markers extracted during the acute post-stroke phase.^26,28–32^ While most existing literature highlights statistically significant correlations between EEG patterns and changes in clinical scales, only a limited number of studies have explored the data-driven predictive capabilities of these markers, particularly concerning the prediction of UE recovery using machine-learning algorithms.^21,33–37^ To our knowledge, none of the previous investigations have validated whether the predictive power of identified EEG metrics is non-redundant in relation to the information already conveyed by the initial FMAUE through the PRR.

For these reasons, the primary objective of this study was to develop a machine-learning framework to predict motor recovery after stroke, specifically in terms of FMAUE scores, using resting-state EEG data. The specific aims of this work were to (i) validate this approach with a dataset of acute stroke patients, where accurately forecasting the evolution of their condition is crucial for optimizing recovery strategies; (ii) assess whether the framework could be generalized to subacute patients; (iii) investigate whether subacute EEG data could enhance the predictive performance for acute datasets; and (iv) determine whether predictive electrophysiological features of motor recovery change across post-stroke phases.

To achieve these goals, we designed and validated a machine-learning pipeline to identify optimal EEG features that complement FMAUE scores in the longitudinal evaluation of stroke survivors. At each stage, we aimed to pinpoint critical EEG features by analyzing a comprehensive dataset of spectral and connectivity measures extensively documented in the literature. The objective was to determine the most effective combination of features that maximized the algorithm's predictive accuracy for future FMAUE score and assess whether these features were both electrophysiologically meaningful and consistent across acute and subacute datasets.

## II. RESULTS

**FIG. 1.**
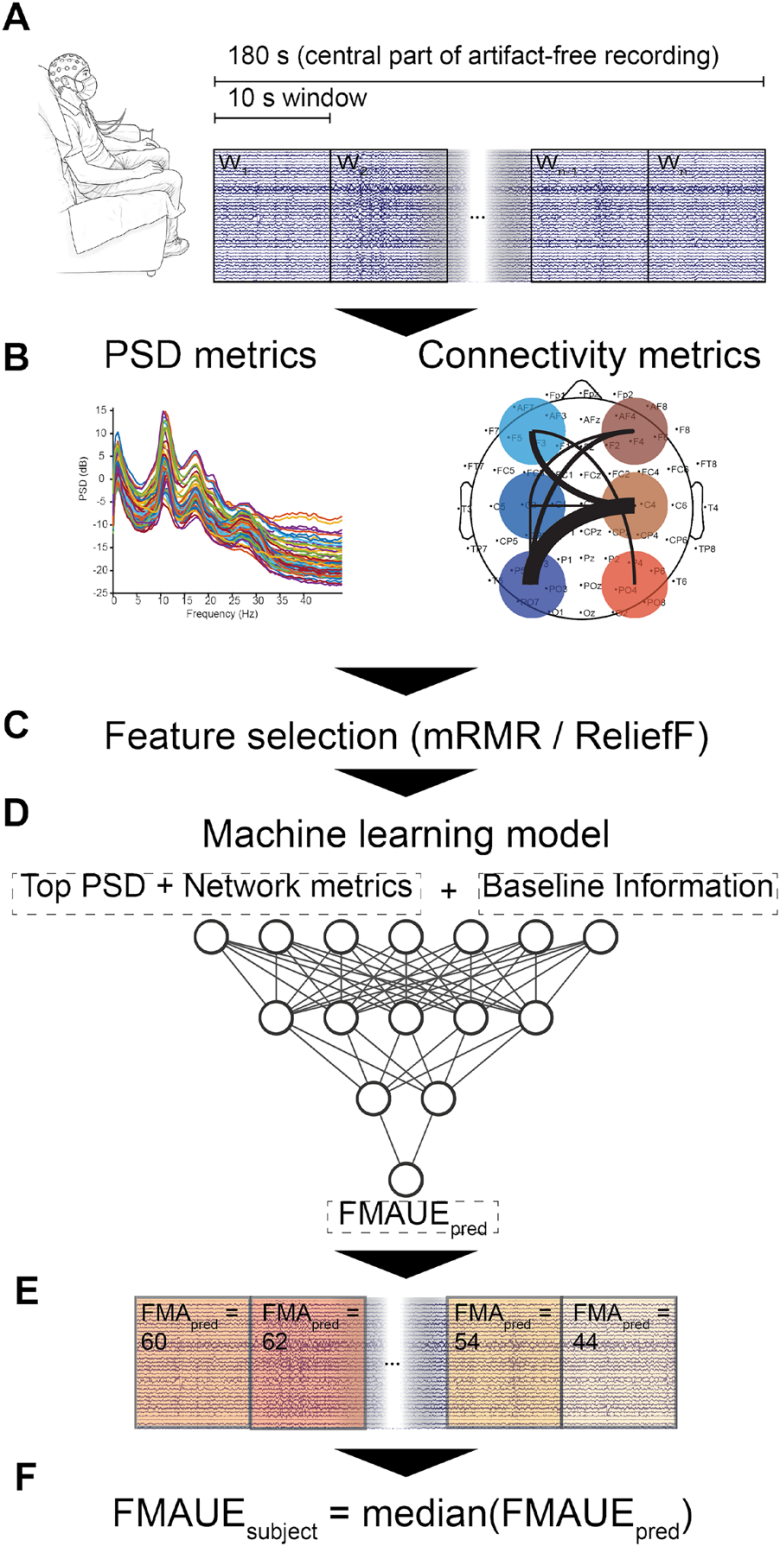
Summary of the applied methodology. (a) EEG is collected at rest and divided into 10 s windows. Only the central 180 s of signal are retained. (b) Metrics are extracted from the power spectrum of the window (left) and from their connectivity patterns, as measured by the imaginary part of the coherency metric. (c) From the entire set of features, the most relevant to the regression task are selected based on the ReliefF and mRMR algorithms. (d) Selected features are used to train *StrokeRecovNet*, an artificial neural network with an optimized number of hidden layers and neurons per layer. (e) The machine-learning model predicts a value of FMAUE for each window of each patient's signal. (f) The estimated prediction of the FMAUE for each subject is obtained by computing the median value of FMAUE among windows.

The proposed machine-learning pipeline, named *StrokeRecovNet*, allowed merging the clinical information of the patient with estimates of various resting-state EEG metrics extracted from overlapping 10-s windows of signal, with the goal of predicting the value of FMAUE at a follow-up visit (T1), performed approximately 3 months after the baseline ( $\text{FMAU}E_{T1}$), starting from a single baseline evaluation. A summary of the proposed model is displayed in Fig. 1. First, the pre-processed EEG signal (see Sec. IV C) was segmented into 10-s windows. From each window, descriptive EEG metrics were extracted, pertaining to spectral content, brain symmetry, connectivity, and network domains (see Sec. IV D). These features were concatenated with the clinical information of the patient [baseline FMAUE ( $\text{FMAU}E_{T0}$), time since stroke (days), and time to second evaluation (days)] and used to train a feed-forward fully connected neural network for regression, with the goal of estimating the $\text{FMAU}E_{T1}$ of the patient. The neural network hyperparameters (number of layers, number of neurons per layer, and batch size), along with the feature selection algorithm and the degree of overlap between windows of the EEG signal, were optimized in an inner validation fold using a nested Leave-One-Subject-Out cross-validation (LOSO) approach (see Sec. IV F). The model was then tested in terms of median absolute error (MAE) on an outer fold of LOSO cross-validation.

To train and test this model, we used two independent datasets (see Sec. IV A): a cohort of 23 participants in the acute post-stroke phase, collected at the “Azienda Ospedaliera Universitaria Pisana” (AOUP-Acute dataset), and a cohort of 17 participants in the subacute phase, recruited at the “Fondazione Don Gnocchi” in Florence, Italy (FDG-Subacute dataset). Both patient cohorts underwent standard rehabilitative treatment between the baseline assessment and follow-up. To address the first three specific aims of this study, we designed three corresponding experiments. First, to validate the proposed approach on acute stroke patients (aim 1), we trained a model using only the acute dataset (AOUP-Acute; experiment #1). Second, to evaluate the generalizability of the framework to subacute patients (aim 2), we trained a separate model on the subacute dataset (FDG-Subacute; experiment #2). Finally, to investigate whether subacute EEG data could enhance predictive performance for acute patients (aim 3), we trained a model on the combined acute and subacute datasets and tested it exclusively on acute patients (AOUP + FDG-Combined; experiment #3) (see Sec. IV E). Once the models for the three experiments were developed and trained, we aimed at comparing selected features from each model (aim 4) by means of clustering similarity measures (see Sec. IV H).

### A. Acute and subacute patients follow the PRR model

First, we verified that the two patient groups roughly followed the proportional recovery rule (PRR) (see Sec. IV B) and adopted it as our baseline model for later comparison. This simple yet clinically established model served as a benchmark for comparing the predictive power of *StrokeRecovNet*.

As expected, the acute population (AOUP-Acute dataset) followed more closely the PRR model by having a larger range of recovery [ $\Delta \text{FMAU}E_{T1-T0}$ range: 0–47, Fig. 2(a)], while the subacute model did it as well, but with a smaller range of recovery [ $\Delta \text{FMAU}E_{T1-T0}$ range: 0–22, Fig. 2(b)]. We also identified a slightly higher proportion of non-recoverers in the subacute cohort (6/23 for AOUP-Acute, 6/17 for FDG-Subacute). Nonetheless, two clear recovery clusters could be identified: recoverers (REC) and non-recoverers (NO-REC). The absolute error of the PRR for each participant served as a baseline model for comparison with our *StrokeRecovNet*.

**FIG. 2.**
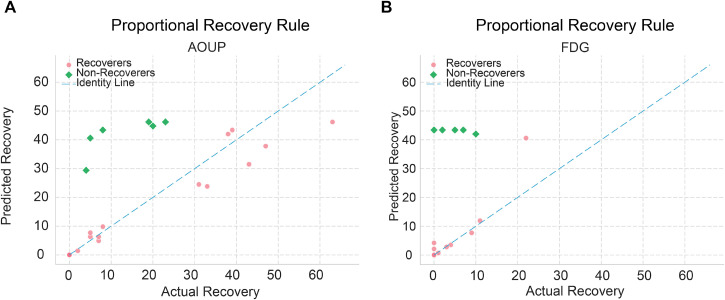
Proportional recovery rule (PRR). (a) PRR in the acute patients dataset. (b) PRR in subacute patients dataset. Pink dots represent patients following the PRR (see Methods), while green squared points represent the non-recoverers. Actual recovery was measured as the difference between FMAUE_*T*1_ and FMAUE_*T*0_. Predicted recovery is the same measure as estimated by the PRR.

### B. Acute EEG data alone do not predict recovery better than PRR

We then investigated whether the proposed architecture can be used to improve the PRR in the acute post-stroke phase. We trained the regression model on the acute dataset only (AOUP-Acute, experiment #1) and checked its performance in predicting FMAUE_*T*1_. For all experiments, we used a nested LOSO cross-validation approach: we trained and optimized the hyperparameters of our network in an inner validation loop, in which one participant's data were left out per iteration. The model with optimal hyperparameters [having the lowest root mean square error (RMSE) on the validation LOSO] was then used to test the prediction performance on the test subject of the outer LOSO loop. The testing process was repeated separately for each subject in the dataset, leading to an unbiased estimate of the performance of the model for each subject.

The optimal hyperparameters selected for each patient in the internal LOSO loop are shown in Table S1.

**FIG. 3.**
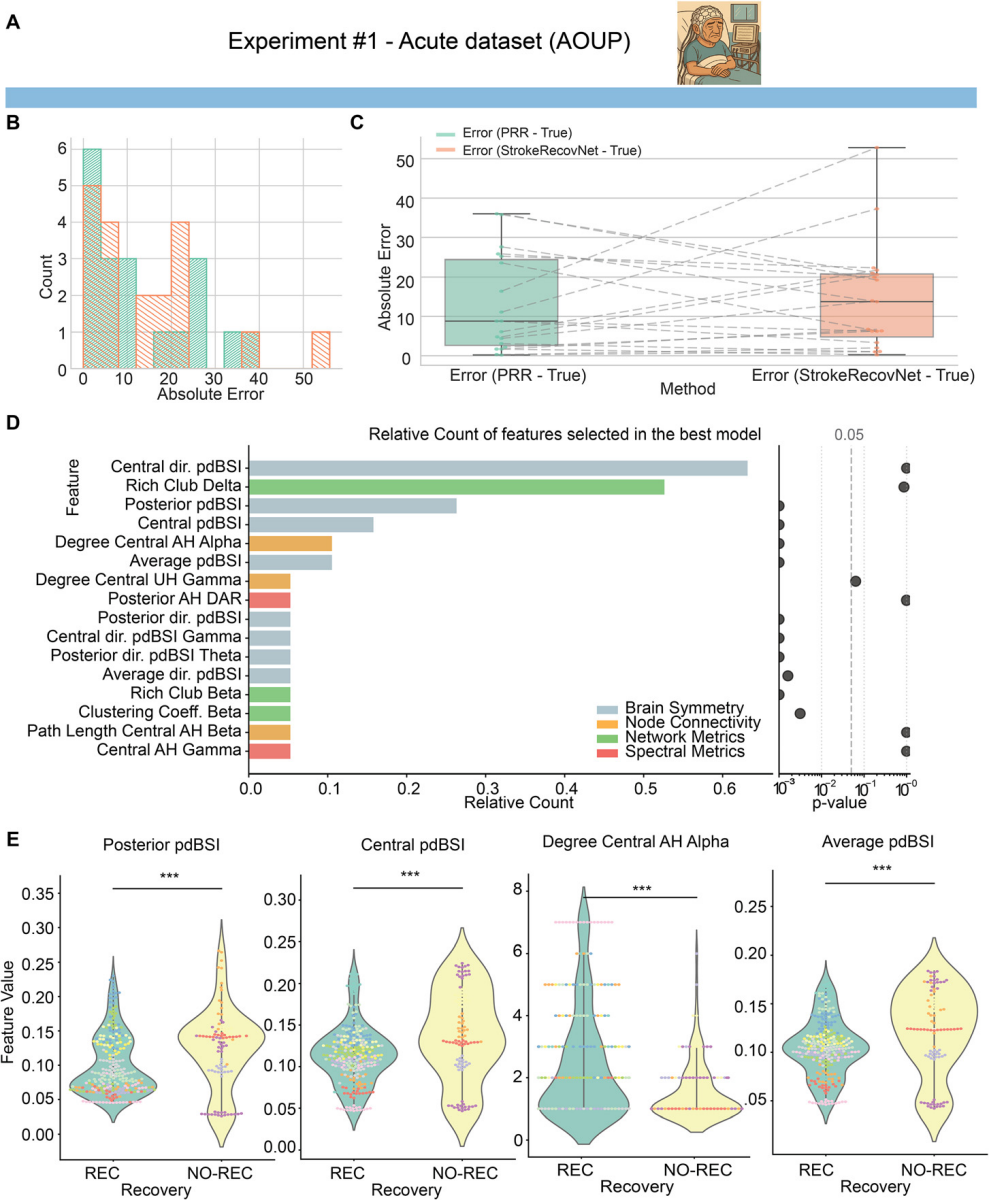
Model predictions based on acute data only. (a) Dataset identifier showing the acute cohort used in experiment #1. This image is repeated in Fig. 5 when the same dataset is used for training. (b) Distribution of errors for the proportional recovery rule (green) and the *StrokeRecovNet* (orange) trained on the AOUP-Acute data only. (c) Boxplots of the absolute error of the predicted $\text{FMAU}E_{T1}$ PRR model (green) and *StrokeRecovNet* (orange). Each point represents the error for a single subject. Dashed lines connect the same subjects between the two models. (d) Left: feature importance barplot based on the frequency of selection in the final model during the LOSO-CV process. Each bar is colored depending on the feature domain it belongs to; right: p-value of the Mann–Whitney test for differences between feature values in the recoverer and non-recoverer groups. Values are lower bounded at 0.001. (e) Violin plots of the distribution of the top four selected significant features (see Methods) in the recoverers (green) and non-recoverers (yellow) groups. Each point represents the value of the feature in a given window of the EEG signal. The color of each point distinguishes different subjects. Statistical significance bars are coded as follows: ^*^0.01 < p < 0.05; ^**^0.001 < p < 0.01; and ^***^p < 0.001.

As shown in Fig. 3(b), *StrokeRecovNet* was able to slightly shift the distribution of prediction errors from two distinct clusters toward one. However, no statistically significant difference was observed between AOUP-Acute model performances and PRR [Fig. 3(c), StrokeRecovNet: 13.74 (16.00), PRR: 8.80 (21.75), all results reported as median (interquartile range), p = 0.908]. Means and standard deviations of the errors are reported in Table S3.

We then aimed at identifying the features that were most relevant in the $\text{FMAU}E_{T1}$ prediction. Each final model, built separately for each subject, selected up to four features from an initial large number of candidate biomarkers, based on their predictive power in the inner LOSO validation. The best models, built separately in an LOSO fashion, selected mostly features related to brain symmetry index: out of the 16 features ever selected in the best-performing model, 8 of them pertained to brain symmetry index [Fig. 3(d), left]. Moreover, the most selected feature was the directional pairwise-derived brain symmetry index (dir. pdBSI) measured over the central areas of the scalp, selected in 62% of the best-performing models. The only relevant exception to the major importance of pdBSI metrics was related to the second most selected feature, which is the network metric of Rich Club in the delta band, selected 53% of the time.

Additionally, we tested whether there were statistically significant differences between REC and NO-REC patients for the features selected in the final models. This analysis aimed at determining whether *StrokeRecovNet* most relevant features captured the electrophysiological characteristics of non-recoverers. Interestingly, the two most selected features did not hold a statistically significant difference between REC and NO-REC, while several significant differences in feature values were found in the less selected features [Fig. 3(d), right]. Specifically, 10 of the 16 selected features exhibited statistically significant differences between REC and NO-REC participants. We focused on the top four selected features that were also statistically significant and visualized them in Fig. 3(e): posterior pairwise-derived brain symmetry index (pdBSI, Mann–Whitney U = 10 051, p < 0.001, Cohen's d = −0.48), central areas pdBSI (Mann–Whitney U = 10 237, p < 0.001, Cohen's d = −0.53), degree of central areas of the affected hemisphere (AH) in the alpha band (Mann–Whitney U = 18 912.5, p < 0.001, Cohen's d = 0.62), and average pdBSI (Mann–Whitney U = 10 180, p < 0.001, Cohen's d = −0.49).

### C. Subacute recovery can be predicted by EEG metrics

We then trained the same network on a dataset of subacute participants (FDG-Subacute dataset, experiment #2).

**FIG. 4.**
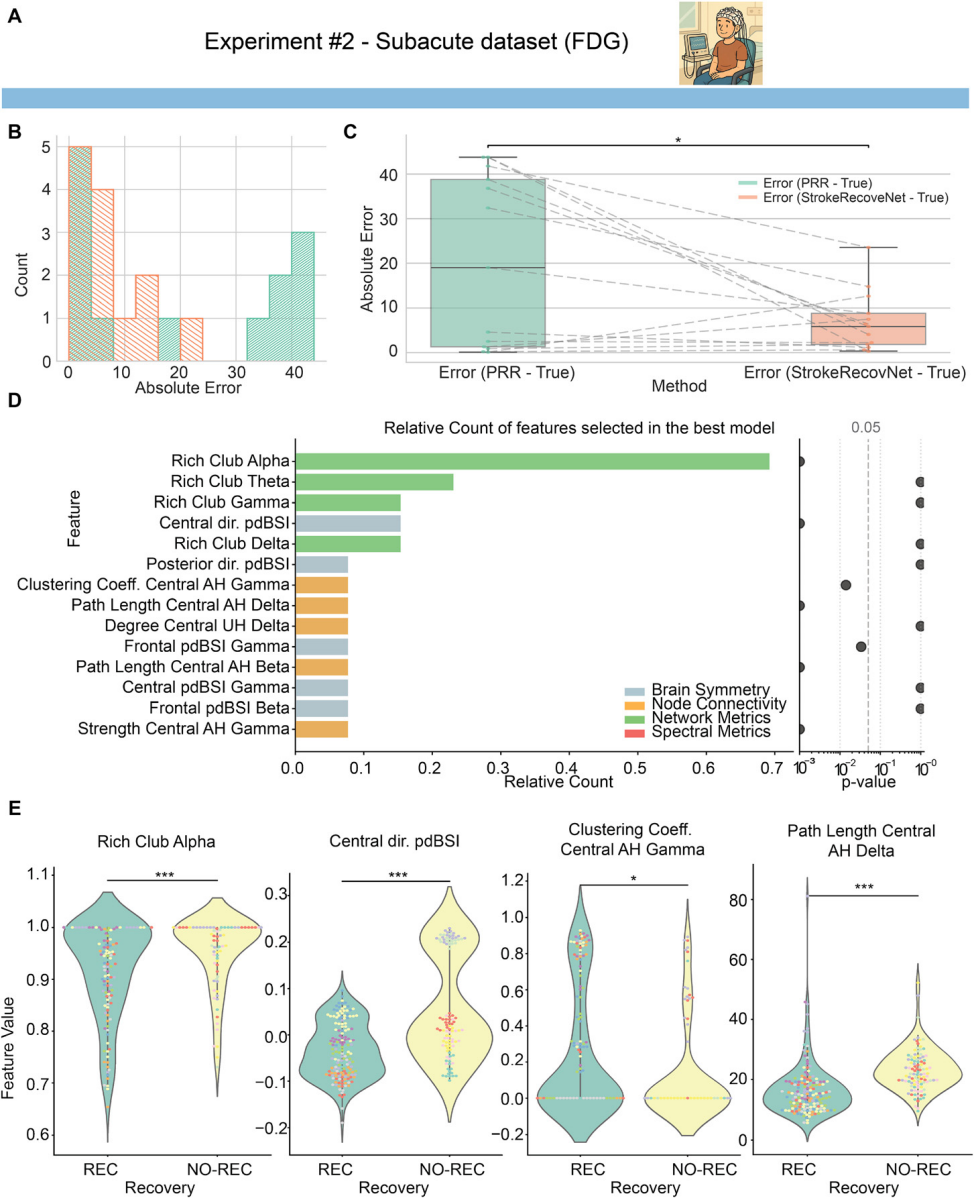
Model predictions based on subacute data only. (a) Dataset identifier showing the subacute cohort used in experiment #2. This image is repeated in Fig. 5 when the same dataset is used for training. (b) Distribution of errors for the proportional recovery rule (green) and the *StrokeRecovNet* (orange) trained on the FDG-Subacute data only. (c) Boxplots of the absolute error of the predicted ${FMAUE}_{T1}$ PRR model (green) and *StrokeRecovNet* (orange). Each point represents the error for a single subject. Dashed lines connect the same subjects between the two models. (d) Left: feature importance barplot based on the frequency of selection in the final model during the LOSO-CV process. Each bar is colored depending on the feature domain it belongs to; right: p-value of the Mann–Whitney test for differences between feature values in the recoverer and non-recoverer groups. Values are lower bounded at 0.001. (e) Violin plots of the distribution of the top four selected significant features (see Methods) in the recoverers (green) and non-recoverers (yellow) groups. Each point represents the value of the feature in a given window of the EEG signal. The color of each point distinguishes different subjects. Statistical significance bars are coded as follows: ^*^0.01 < p < 0.05; ^**^0.001 < p < 0.01; and ^***^p < 0.001.

By following the same training–validation procedures as in experiment #1, we achieved much better performance on the FDG-Subacute dataset. Specifically, *StrokeRecovNet* significantly outperformed the PRR model in predicting $\text{FMAU}E_{T1}$ in the subacute group [absolute error, *StrokeRecovNet*: 5.85 (6.97), PRR: 19.00 (37.5), permutation test: median difference = −13.57, p = 0.023, Figs. 4(b) and 4(c)].

Differently from experiment #1, selected features were mostly pertaining to the network and node connectivity domains [Fig. 4(d)]: out of the 12 total selected features, three belonged to the network domain and five to the node connectivity one. Importantly, the first selected feature (Rich Club in the alpha band) was selected 72% of the time, far outperforming the second most important feature (Rich Club in the theta band, selected 26% of the time).

When looking at statistical differences in distinguishing REC and NO-REC, we observed several significant patterns: seven out of the 12 features significantly differed between the two groups. The first four significant features [Fig. 4(e)] were Rich Club in the alpha band (Mann–Whitney U = 6131, p = 0.001, Cohen's d = −0.504), dir. pdBSI in the central areas (Mann–Whitney U = 4682, p < 0.001, Cohen's d = −1.035), clustering coefficient of the central node of the AH in the gamma band (Mann–Whitney U = 10 361, p = 0.014, Cohen's d = 0.377), and path length of the central node of the AH in the delta band (Mann–Whitney U = 3203, p < 0.001, Cohen's d = −0.915).

### D. Acute recovery prediction improves by including supporting subacute data

Finally, we tested whether training on the combined AOUP-Acute and FDG-Subacute datasets could improve the prediction of the acute data. Hence, we trained the *StrokeRecovNet* on the combination of the two datasets and tested its performance on the acute patients with the same nested LOSO approach (AOUP + FDG-Combined, experiment #3).

**FIG. 5.**
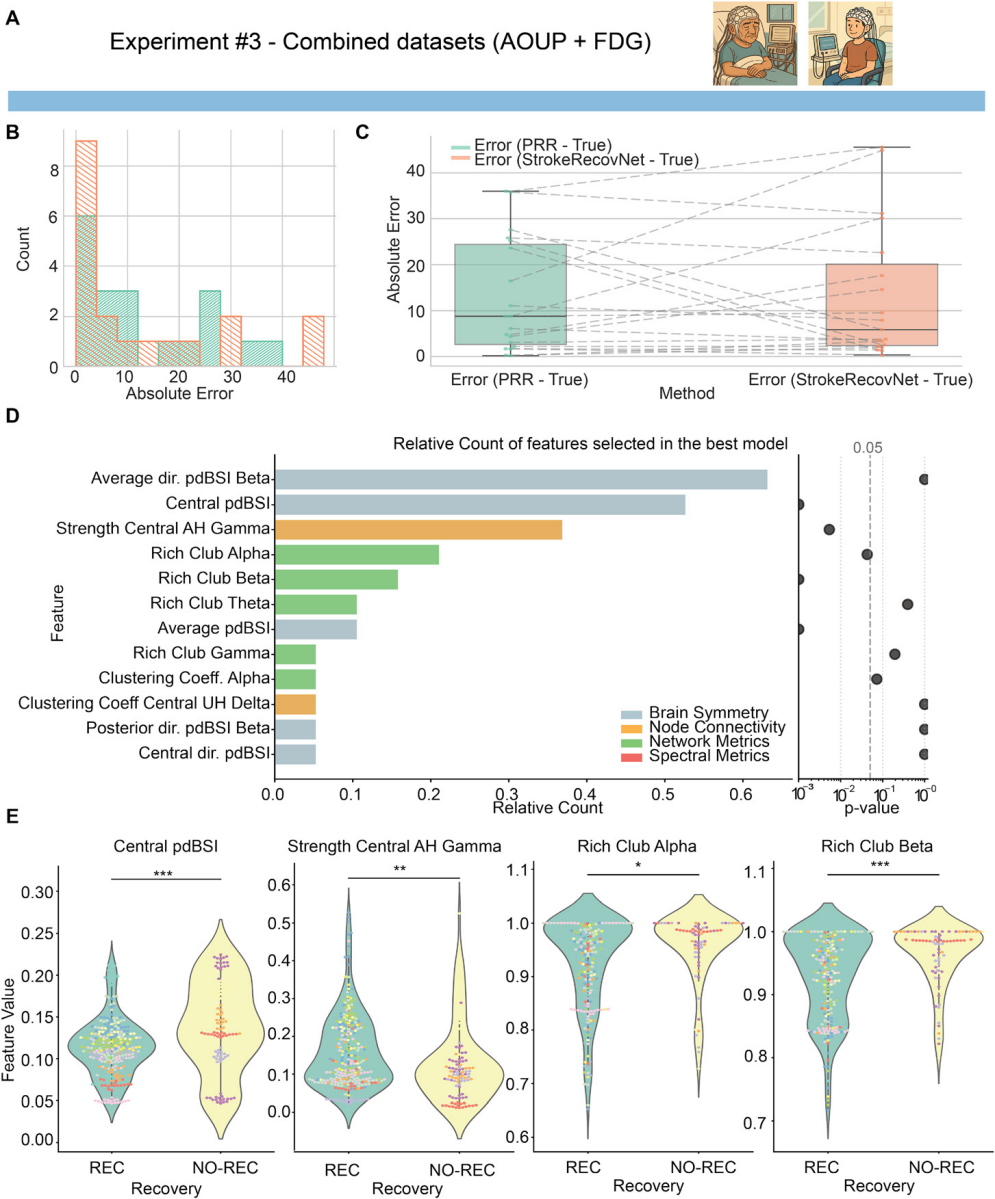
Model predictions based on the combined dataset. (a) The dataset identifiers indicate that the subacute and acute cohorts were used for training (see also experiments #1 and #2). (b) Distribution of errors for the proportional recovery rule (green) and the *StrokeRecovNet* (orange) trained on the AOUP-Acute and FDG-Subacute data. (c) Boxplots of the absolute error of the predicted $FMAUE_{T1}$ PRR model (green) and *StrokeRecovNet* (orange). Each point represents the error for a single subject. Dashed lines connect the same subjects between the two models. (d) Left: feature importance barplot based on the frequency of selection in the final model during the LOSO-CV process. Each bar is colored depending on the feature domain it belongs to; right: p-value of the Mann–Whitney test for differences between feature values in the recoverer and non-recoverer groups. Values are lower bounded at 0.001. (e) Violin plots of the distribution of the top four selected significant features (see Methods) in the recoverers (green) and non-recoverers (yellow) groups. Each point represents the value of the feature in a given window of the EEG signal. The color of each point distinguishes different subjects. Statistical significance bars are coded as follows: ^*^0.01 < p < 0.05; ^**^0.001 < p < 0.01; and ^***^p < 0.001.

While the combined model tested on AOUP-Acute data was not significantly better than the PRR [permutation test: median difference = −2.92, p = 0.904, Figs. 5(b) and 5(c)], the median absolute error of the model trained on the two combined datasets (absolute error StrokeRecovNet: 5.87 [17.71]) overcame that of the model trained on AOUP-Acute data only (13.74 [16.00], see Sec. II B) and improved the PRR itself (absolute error PRR: 8.80 [21.75]). Interestingly, the model from experiment #3 performed analogously to a model trained on a fixed feature set (see the supplementary material) based on the findings of Saes *et al.*^26,30^ Additionally, the combined model also performed similarly to a model trained solely on FDG-Subacute data and tested on the AOUP-Acute dataset (see the supplementary material).

When looking at the most selected features, a mixture of the two previous models emerged: out of the 12 selected features across the LOSO loop, five of them belonged to brain symmetry metrics, five to network metrics, and two to node connectivity. The top two selected features (average dir. pdBSI and central pdBSI) were selected 63% and 55% of the time, respectively.

Statistically, only five of those features distinguished significantly between REC and NO-REC groups [Fig. 5(d), right]. The top four most selected were central pdBSI (Mann–Whitney U = 10 237, p < 0.001, Cohen's d = −0.533), Rich Club in the beta band (Mann–Whitney U = 11 766, p = 0.028, Cohen's d = −0.358), and dir. pdBSI in the central areas (Mann–Whitney U = 22 975, p < 0.001, Cohen's d = 1.117).

### E. Predictive features change between acute and subacute stroke

In our three experiments, we trained the same neural network structure to learn predictive feature representation of acute and subacute motor recovery. To assess the reproducibility and coherence of multi-domain EEG-derived metrics across different cohorts, we performed hierarchical clustering and consistency scoring analyses (see Sec. IV H for details).

**FIG. 6.**
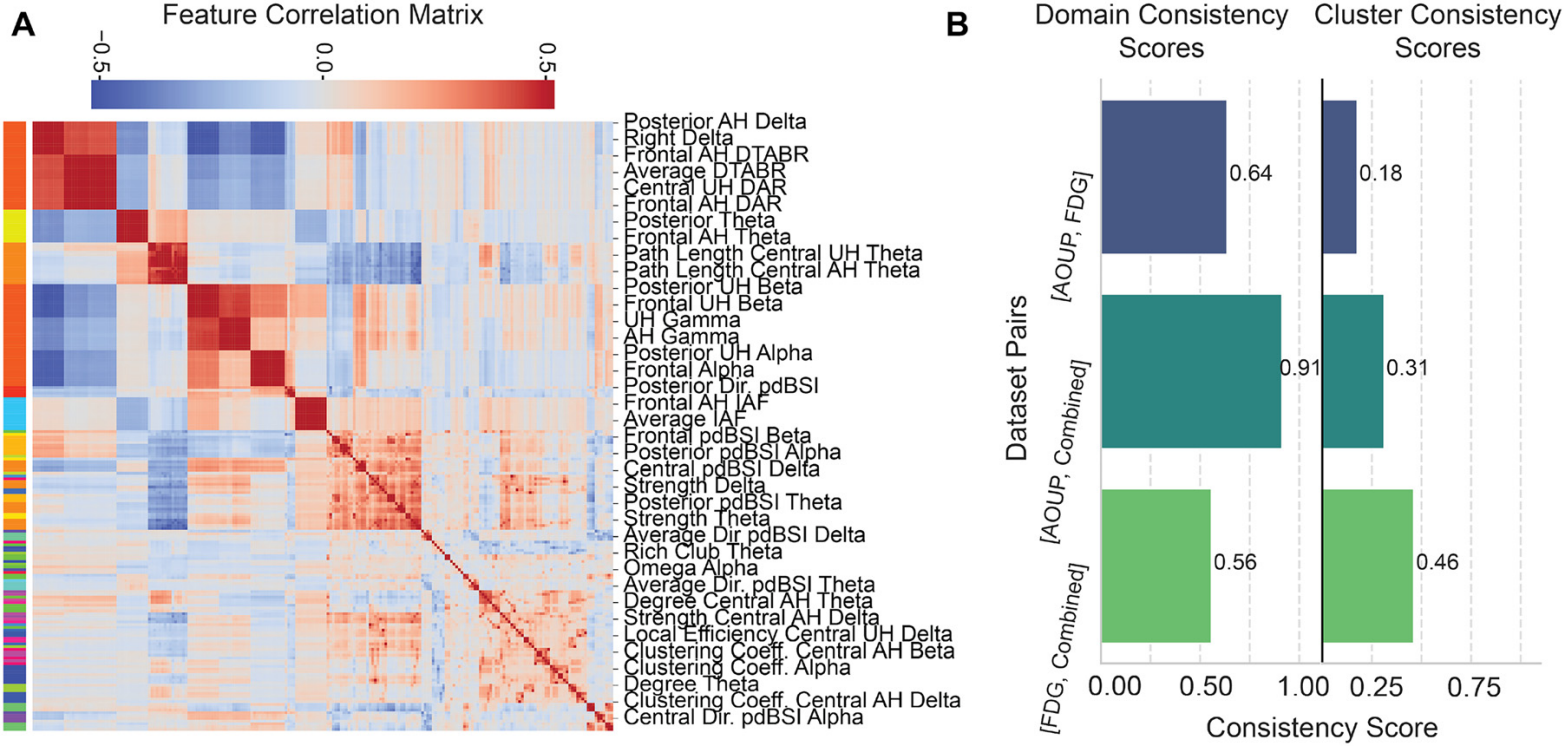
Feature consistency across models. (a) Correlation matrix in the acute dataset, sorted based on hierarchical clustering (see Methods). Each color in the bar on the left represents a cluster of features obtained through hierarchical clustering. The color inside the heatmap represents the Pearson correlation between pairs of features. (b) Consistency scores between pairs of models trained on different datasets. On the left, the consistency score is based on predetermined domains. On the right, the consistency score is based on clusters defined on hierarchical clustering.

Figure 6(a) presents the hierarchical clustering heatmap of all extracted EEG features across datasets. The dendrogram and corresponding correlation matrix highlighted distinct clusters of features that covaried across subjects, forming consistent data-driven domains. Notably, features related to delta power and connectivity (e.g., posterior AH delta, frontal AH DTABR) clustered separately from alpha- and beta-related metrics (e.g., frontal alpha, posterior unaffected hemisphere alpha), indicating potential domain-specific grouping. Additionally, topological network features (e.g., degree, clustering coefficient, and rich club organization) formed cohesive clusters distinct from spectral power and frequency-based measures, suggesting functional segregation of metric types.

To quantify the robustness of these feature groupings across datasets, we computed two consistency scores: domain consistency and cluster consistency [Fig. 6(b)]. Domain consistency, which reflects the reproducibility of predefined feature domains, was highest between the AOUP-Acute and the AOUP + FDG-Combined datasets (0.91) and moderate between AOUP-Acute and FDG-Subacute (0.64) and FDG-Subacute and AOUP + FDG-Combined (0.56). This suggests that the domain-level structure of EEG metrics is largely preserved across cohorts, especially when aggregated.

Cluster consistency, which measures the reproducibility of fine-grained feature clusters extracted with a data-driven approach, showed moderate values across all dataset pairs, with the highest score observed between the FDG-Subacute and the AOUP + FDG-Combined dataset (0.46). However, the lowest cluster consistency score (0.18) was obtained between the acute and subacute datasets trained independently. The AOUP + FDG-Combined dataset model shows a higher consistency both with AOUP-Acute (0.31) and FDG-Subacute (0.46), indicating that shared feature patterns are acquired when training the combined model.

## III. DISCUSSION

In this study, we introduced and validated a machine-learning pipeline named *StrokeRecovNet* (Fig. 1), designed to predict FMAUE scores at follow-up using resting-state EEG data collected during the acute or subacute post-stroke phases. Our data-driven approach underscores that meaningful predictive features can be identified from EEG recordings after a stroke, offering a promising avenue for predicting UE recovery. At the core of our methodology is a feed-forward, fully connected neural network tailored for regression tasks, with carefully optimized layer configurations and feature selection.

We focused specifically on forecasting UE recovery during the acute and subacute post-stroke phases. Our primary objective was to build a predictive model capable of making accurate forecasts very early after stroke onset, as early prediction of motor outcomes could facilitate the optimization of rehabilitation protocols and capitalize on the brain's heightened plasticity during this period, ultimately enhancing recovery potential.^38^

Notably, our results show that *StrokeRecovNet* significantly outperformed the conventional proportional recovery rule (PRR) (Fig. 2) in predicting $\text{FMAU}E_{T1}$ scores in the subacute dataset (Fig. 4). In the case of acute data, training the model on acute-phase data alone did not improve performance over PRR (Fig. 3); however, performance markedly improved when subacute data were incorporated during training (Fig. 5). This combined training enabled the network to learn a multi-phase, multi-center representation of the data (Fig. 6), proving effective across two independent datasets. These findings highlight the superiority of machine-learning models leveraging EEG-derived metrics compared to those relying solely on $\text{FMAU}E_{T0}$.

Although the acute and subacute datasets differ in their distribution of recovery levels as highlighted by PRR patterns, our analyses indicate that this imbalance alone does not account for the observed performance differences. In particular, the subacute EEG features contribute to phase-specific physiological information that improves prediction in acute patients when the datasets are merged. This suggests that the model benefits from complementary electrophysiological signatures more than recovery level (i.e., PRR) alone.

While previous models for predicting UE recovery, such as PREP2, have been proposed in the literature, they typically rely on initial impairment scores combined with measures of corticospinal tract (CST) integrity.^9^ Although EEG measures and CST integrity reflect distinct aspects of post-stroke brain function, studies have reported potential correlations between the two.^21,39^ Given that CST integrity is often assessed using costly techniques, such as detecting motor-evoked potentials via transcranial magnetic stimulation or through diffusion-weighted imaging, EEG offers a more accessible and cost-effective alternative.

### A. Predictive performance of *StrokeRecovNet* in acute and subacute phases

Interestingly, when the model is trained exclusively on acute-phase data, its average median absolute error (MAE) does not surpass that of the PRR. In contrast, training on subacute data results in significantly better performance compared to PRR. This discrepancy may stem from several interrelated factors. Most notably, acute and subacute patients may exhibit distinct neural signatures due to ongoing plastic changes in the post-stroke recovery process. These evolving brain dynamics may limit the predictive power of electrophysiological metrics when only acute data are considered. Additionally, inherent differences in EEG recordings may influence model performance. Despite our efforts to standardize the datasets by applying identical recording protocols, preprocessing pipelines, and feature extraction procedures, we anticipated differences in signal-to-noise ratio, particularly in the acute cohort. This is likely due to the increased complexity of recording EEG in acute stroke survivors and may contribute to the lower predictive performance observed for the acute-only model.

To address these limitations, we combined the acute and subacute datasets into a unified training set in experiment #3. This approach mitigated dataset-specific biases and led to performance improvements, even in acute-phase predictions. These results underscore the advantages of increasing both the diversity and volume of training data. Training with the subacute cohort enabled the model to more effectively estimate network weights and to learn a more comprehensive representation of the predictive features associated with stroke recovery.

These observations are further supported by the optimal hyperparameters identified in each experiment, as reported in Table S1. In experiments #1 and #2, where training was conducted separately on acute and subacute data, the preferred hyperparameters typically included a high overlap of 7.5 s between analysis windows, likely a strategy to compensate for the limited dataset size. In these experiments, the ReliefF algorithm was commonly selected for feature selection. However, in experiment #3, the expanded training set enabled effective learning even with lower window overlaps and smaller batch sizes, enhancing the model's generalization capacity. Moreover, the mRMR (minimum redundancy maximum relevance) feature selection method emerged as optimal, likely due to its ability to identify non-redundant yet informative features. These trends suggest that further increasing the training data could lead to more stable models with consistent hyperparameter configurations across participants.

Although the performance of our model does not yet constitute a perfect prediction of $\text{FMAU}E_{T1}$, the results are highly promising in light of the minimal clinically important difference (MCID) for FMAUE, which ranges from 4.25^40^ to 12.4^41^ according to prior studies. Thanks to the rigor of our nested LOSO cross-validation framework, we ensured that no data leakage occurred between training and testing sets. Consequently, we can reasonably expect the generalization error of *StrokeRecovNet* to approximate the error observed in our test evaluations.

### B. Feature relevance divergence in acute and subacute patients

When analyzing the features selected by the optimal models, several intriguing patterns emerged. In experiment #1 [Figs. 3(d) and 3(e)], *StrokeRecovNet* predominantly selected spectral features, particularly those related to brain symmetry. Notably, the most frequently selected feature in predicting outcomes from acute data was the directional brain symmetry index (dir. pdBSI) in central scalp regions, primarily corresponding to the sensorimotor cortex. This finding provides strong physiological validation, as it was derived entirely through data-driven selection. In addition to symmetry metrics, the only network-level feature selected was the Rich Club coefficient in the delta band, a marker of how densely connected the network's hub nodes are to each other. The affected hemisphere also played a significant role in acute-phase predictions: spectral metrics such as the posterior affected hemisphere (AH) delta/alpha ratio (DAR) and central node connectivity features were frequently selected in the best-performing models.

In contrast, experiment #2 [Figs. 4(d) and 4(e)] revealed a shift in the types of features relevant to prediction during the subacute phase. Rather than symmetry indices, the model placed greater importance on network-level and nodal connectivity metrics. Specifically, we observed that participants with good recovery (REC) exhibited lower Rich Club coefficients across several frequency bands compared to non-recoverers (NO-REC). This may reflect a more preserved or reorganized network structure, enabling more efficient connectivity regulation. While such patterns have not been reported in previous EEG studies, similar observations in MRI research have linked higher numbers of Rich Club regions to greater neurological impairment post-stroke.^42^ Once again, regions over the sensorimotor cortex emerged as critical, with several local connectivity features contributing to the model's predictions. For example, path length in the delta band within the central region of the affected hemisphere was increased in the NO-REC group, suggesting disrupted communication from or to this area.

Interestingly, both experiments #1 and #2 demonstrated notable consistency in the selection of robust predictors across LOSO cross-validation folds. In the acute dataset, symmetry-related features were repeatedly selected, while in the subacute dataset, network connectivity metrics showed the highest stability. Although specific frequency bands and spatial locations varied across folds, these differences likely stem from variation in patient inclusion across training sets. Overall, this behavior supports the idea that, despite minor fluctuations in selected features, the models consistently identified the same domains of predictive relevance.

Overall, our findings are consistent with previous work in this field. EEG prediction of post-stroke neurological deficit recovery has been widely established in prior research.^24,31,43^ Among the various EEG-derived markers used in this context, the delta-to-alpha ratio (DAR) has been one of the most consistently associated with neurological impairment, as well as with global functional prognosis in the subacute context for left-hemisphere strokes.^44^ Apart from a minor importance in experiment #1, we did not find DAR to be predictive of motor recovery, in line with the few studies investigating upper extremity motor recovery biomarkers.^30,45–48^ Consistent with our findings, these studies highlight the prognostic value of brain symmetry and interhemispheric connectivity. In contrast, studies on subacute stroke populations tend to emphasize the role of connectivity and network-level features in predicting motor outcomes,^21,49,50^ with fewer highlighting symmetry-related measures,^51^ as we found in experiment #2. Additionally, these results are consistent with what was previously found by our group,^52^ indicating a differential evolution of spectral metrics throughout different phases of stroke recovery.

Our results also point to a coherent physiological interpretation of why different EEG features emerge as predictive at different stages and why they differentiate recoverers from non-recoverers. In the acute phase, the prominence of delta and alpha abnormalities, together with marked interhemispheric asymmetry (pdBSI and directional pdBSI), reflects the direct physiological consequences of ischemia. Delta activity is typically generated by dysfunctional or deafferented tissue, whereas alpha rhythms, largely generated in cortical layer IV, diminish proportionally to cortical lesion burden.^53–55^ Thus, the spectral profile observed in the acute stage maps naturally onto underlying lesion topology: more delta and reduced alpha indicate more extensive cerebral dysfunction or cortical injury, and higher directional asymmetry indicates a clear dominance of the intact hemisphere over the lesioned one, reflecting acute disruption of interhemispheric balance.^27,29^ In contrast, beta and gamma oscillations emerge only when local cortical microcircuitry and cortico-cortical pathways are relatively preserved;^56–58^ these higher-frequency rhythms require intact inhibitory–excitatory coupling and therefore tend to reappear as the brain transitions into the subacute period. Their predictive value in the combined- and subacute-phase models aligns with evidence that beta oscillations support sensorimotor integration and corticospinal integrity, while gamma reflects short-range cortical reinstatement and local processing capacity.^59,60^ Directional pdBSI complements this view by indicating not only the magnitude but also the direction of hemispheric imbalance, thereby capturing whether slow-wave excess or fast-rhythm suppression is driving functional asymmetry at each time point.

The differences observed between patients who recovered and those who did not similarly reflect these time-dependent mechanisms. Patients with better outcomes showed more preserved alpha activity [in terms of connected areas, Fig. 3(e)] and increased symmetry over the sensorimotor and occipital areas (posterior and central pdBSI). This may indicate that recoverers in the acute stage may be characterized by markers of limited structural damage and less extensive disconnection. Complementarily, in the subacute phase, recoverers were characterized by more adaptive network configurations, such as reduced pathological synchronization in the delta band, reduced Rich Club behavior in the alpha band, and increased clustering in the gamma band. Subacute recoverers are hence characterized by a well-interconnected network function, with reduced pathological coupling and increased physiological (i.e., gamma band activity) oscillations. Together, these results suggest that stroke recovery is supported by distinct electrophysiological signatures at different time points: early spectral abnormalities reflecting injury burden and later network-based features indexing the brain's capacity for functional reintegration.

Lesion characteristics are known to modulate post-stroke electrophysiology, particularly when cortical territories or hub regions are involved, as these areas play a central role in oscillatory dynamics and interhemispheric coordination.^61,62^ For this reason, we examined whether the model prediction error was systematically related to lesion location or lesion extent (see the supplementary material). Our analyses did not reveal significant associations between prediction error and lesion topography (i.e., cortical vs subcortical involvement, number of affected areas). This absence of systematic effects suggests that mispredictions are unlikely to arise from specific anatomical profiles and that our model does not disproportionately fail in patients with particular lesion patterns. Instead, errors may reflect more diffuse, network-level factors that are not captured by coarse structural descriptors alone, consistent with prior evidence that global diaschisis and connectivity disruption can outweigh focal lesion characteristics in shaping recovery trajectories.^63^

### C. Combined training captures common patterns of motor recovery

In experiment #3 [see Figs. 5(d) and 5(e)], combining the acute and subacute datasets led to the selection of a feature set that integrates elements from both previous experiments. Specifically, while brain symmetry indices remained the most prominent predictors, consistent with findings from experiment #1, several network metrics, previously observed in experiment #2, also emerged among the selected features. These additional metrics contributed to improved prediction performance on acute data, suggesting that training on the combined dataset enables the model to learn more generalizable patterns of recovery shared across different post-stroke phases. While electrophysiological activity evolves rapidly after stroke, several key markers of recovery, specifically interhemispheric asymmetry and connectivity reorganization, are present across both phases. Subacute EEG, acquired in a more stable physiological state, likely provides cleaner examples of these mechanisms, allowing the model to generalize them back to acute data.

From a frequency-domain perspective, experiments #1 and #2 primarily identified features associated with broadband activity, as well as with delta and alpha bands, commonly implicated in stroke recovery prognosis.^24,51^ However, in experiment #3, features from higher-frequency bands, such as beta and gamma, also appeared among the most informative predictors. This finding may guide future investigations toward exploring these higher frequencies, which may contain complementary yet clinically relevant information for predicting motor recovery. In particular, the gamma band, though often excluded from EEG analyses due to its lower signal-to-noise ratio, is being increasingly recognized for its relevance in the pathophysiology of various neurological disorders in both animal models^64^ and humans.^65^

Across all three experiments, we observed that the features most frequently selected by the model were not always those showing the strongest univariate statistical differences between REC and NO-REC groups. This observation likely reflects two key factors. First, *StrokeRecovNet* is designed to capture complex, non-linear interactions both between features and with the outcome variable, as recently demonstrated in a similar work with animal models.^33^ Second, the model predicts a continuous outcome, FMAUE at follow-up, rather than a binary classification of recovery status. As a result, features that are significant in discriminating between REC and NO-REC groups may not necessarily be optimal in the multivariate regression context of *StrokeRecovNet*.

### D. Toward a multi-phase, multi-center prediction of stroke recovery using EEG

In experiment #3, we demonstrated that heterogeneous datasets can be effectively leveraged to enhance the prediction of acute stroke recovery. This is a key strength of our model, as it highlights its flexibility and provides insights into the physiological traits that are most predictive at different stages of stroke recovery. While subacute prediction yielded strong results on its own, predicting recovery in the acute phase proved more challenging. Notably, incorporating subacute data improved acute prediction performance, underscoring the value of cross-phase data support.

As shown in Fig. 6(b), this improvement is not solely attributable to the increased number of training samples. Rather, the model trained on the combined datasets developed a different internal representation of the relevant features. Specifically, we observed that this model learned a blended representation of the acute and subacute data, as evidenced by higher cluster consistency scores in AOUP-Acute vs Combined and FDG-Subacute vs Combined comparisons than in AOUP-Acute vs FDG-Subacute. While this finding may seem intuitive, the key takeaway is that the model's improved performance on acute data stems from this enriched representation, suggesting the possibility to capture the complexities of acute recovery more effectively than training on acute data alone. Moreover, our *StrokeRecovNet* performs similarly to a model trained solely on a fixed set of known predictive features (see the supplementary material and Supplementary Table S4). This confirms that *StrokeRecovNet* is able to automatically identify the most relevant features from a large pool of candidate biomarkers. It could therefore be used as a tool to test novel EEG biomarkers in the future.

We hypothesize that this mixed representation, which incorporates both connectivity and network metrics, as well as spectral and brain symmetry features, more accurately reflects the multifaceted nature of recovery in acute patients. Furthermore, the fact that our datasets originate from different centers reinforces the robustness and generalizability of our pipeline, not only across post-stroke phases but also across varying acquisition environments.

Cross-dataset analyses (see Supplementary Table S7) further showed that features learned in the subacute phase transfer relatively well to acute data, whereas the reverse does not hold. This asymmetry indicates that the electrophysiological markers of recovery differ across phases and supports the need for a combined training set that allows the network to learn features stable across recovery trajectories.

Moreover, the timing of the follow-up assessment varied across participants, particularly in the AOUP cohort. Because time-to-follow-up was included as an input feature, the model could account for differences in recovery windows and adapt predictions accordingly. This design choice likely enabled the network to capture both the rapid early improvements that dominate the first months post-stroke and the smaller, gradual changes observed in later subacute assessments. The merged dataset therefore exposed the model to a broader spectrum of recovery trajectories, which may explain its improved performance across cohorts.

These findings lay the groundwork for extending our approach by integrating data from multiple centers into a unified predictive framework, aiming to maximize both performance and generalizability. Incorporating federated learning techniques could further enhance this framework by enabling collaborative model training across institutions without sharing sensitive patient data, thereby preserving privacy and complying with regulatory constraints. Importantly, our results also support the feasibility of using subacute data to train models for acute recovery prediction. This approach could help overcome the practical challenges of collecting large acute-phase datasets, a recognized obstacle in stroke research.^19^ By enabling the use of available subacute data, our method opens the door to training more complex and powerful models without sacrificing early prediction capabilities.

Our findings can also be contextualized within recent multimodal approaches that combine EEG with additional data modalities or task-based paradigms. Several studies have integrated resting-state EEG with kinematic assessments to capture electrophysiological differences underlying distinct recovery trajectories.^52,66,67^ Other EEG-based work has demonstrated that action-observation interventions enhance mu-desynchronization, underscoring the contribution of sensorimotor resonance mechanisms to motor relearning.^68–70^ Complementary evidence from fMRI highlights that favorable outcomes depend on the restoration of interhemispheric connectivity and the recruitment of distributed premotor–parietal networks.^22^ At the structural level, disconnection-based machine-learning models have predicted motor outcome by quantifying lesion-network disruptions in key white-matter pathways.^71^ Together, these studies illustrate both the relevance of EEG–motor relationships in stroke recovery^72^ and the potential of integrating multimodal information into predictive frameworks.^73^

Compared with these multimodal or anatomically grounded approaches, our method relies solely on resting-state EEG, providing a low-cost and scalable tool suitable for routine clinical deployment. Nonetheless, EEG carries well-known limitations—including susceptibility to artifacts, dependence on signal quality, relatively low spatial resolution, and challenges in source localization. Future work integrating EEG with structural connectivity measures or kinematic features may help mitigate these constraints, improve mechanistic interpretability, and ultimately enhance prediction performance.

Despite the presence of a subset of participants for whom predictions were worse than those from the PRR model, our diagnostic analyses did not reveal systematic associations with stroke severity, lesion topography, follow-up timing, or demographic factors. This suggests that these underperforming cases do not reflect a consistent model bias but rather heterogeneous sources of variability that remain unresolved at the current sample size. The only factor consistently linked to higher prediction errors was EEG preprocessing quality in the FDG-Subacute cohort, indicating that degraded electrophysiological signals can hinder the extraction of reliable neurophysiological markers. Taken together, these findings highlight that while the model generalizes well across most patients, a small proportion remains inherently difficult to predict based on the currently available features, echoing the challenges previously reported for classical PRR-based approaches. This variability is clinically relevant, as the ability to identify individuals who deviate from expected trajectories is essential for both personalized prognostication and adaptive rehabilitation planning. Importantly, our architecture was deliberately constrained to a limited set of interpretable features, which facilitates mechanistic insight and enables the use of explainability tools, such as SHAP,^74^ to probe the contribution of specific EEG and clinical markers to individual predictions.

### E. Limitations and future perspectives

The relatively small participant cohort in the two datasets presents a limitation in delivering a fully reliable estimate of the model's performance on unseen data, as well as in assessing the generalizability of the results. We have in this direction shown the feasibility of training the model on two distinct datasets and shown that combining them improves the predictive performance. Nonetheless, the unequal representation of severe and mild strokes across datasets may influence the relative difficulty of the prediction task. Future studies using larger and more balanced cohorts will help clarify the extent to which dataset composition influences model generalizability. Moreover, cross-dataset performance was not uniform, as models trained solely on acute data did not generalize well to subacute patients. This highlights limited transferability of phase-specific electrophysiological features and suggests that larger, multi-phase datasets will be essential to develop models that generalize robustly across clinical contexts.

Another limitation of the present work is the restriction to four input features, imposed by the computational complexity of exhaustive hyperparameter and feature-set optimization under an LOSO framework. Future studies should evaluate models incorporating a larger number of electrophysiological features and assess whether the optimal feature count can itself be learned as a hyperparameter, potentially improving predictive performance and neurophysiological interpretability. Given the large number of candidate biomarkers our network tries to optimize, it would be beneficial to inject prior knowledge about known good biomarkers into the network. In this regard, as different recovery stages may be characterized by distinct electrophysiological signatures, future improvements of the network will explore Bayesian modeling to incorporate prior knowledge about candidate biomarkers into the feature set. This could simplify training and lead to more accurate predictions.

We also acknowledge that our study solely relies on the FMAUE as the metric for motor recovery assessment. While the FMAUE is a widely recognized measure for evaluating UE motor function post-stroke, it is not without limitations, such as the potential for a ceiling effect and its inability to encapsulate the full complexity of motor deficits in stroke patients. Future work will extend the array of predicted metrics and will devise a composite score for a more comprehensive evaluation of motor recovery. Future studies could expand this modeling approach to broader neurological or functional outcomes, such as the NIH Stroke Scale (NIHSS), allowing a more comprehensive characterization of post-stroke recovery.

Additionally, it would be informative to thoroughly explore the influence of potential covariates on our predictions, including factors such as age and lesion location. We made a first attempt in doing so by checking whether the prediction performance of our model was influenced by the cortical involvement of the lesion or the number of anatomical areas involved. However, future studies should explicitly integrate lesion information into the predictive framework to help refine the interpretation of oscillatory patterns found in the EEG and improve prediction performance. By examining the structural–functional relationships between lesions and the identified EEG features, we will provide a more thorough understanding of the predictive model's performance and its applicability to a wider patient population. Future studies using larger and multi-center datasets will also be crucial to identify latent subgroups of “non-predictable” participants and clarify whether distinct phenotypes drive systematic prediction failures, possibly using explanation algorithms such as SHAP values.^74^

This study establishes the groundwork for crafting predictive models of UE recovery following a stroke, extending from the acute post-stroke phase. This endeavor aims to enhance and personalize existing models of UE motor recovery post-stroke. Subsequent investigations will assess how prediction accuracy and pertinent features evolve when predicting various time points within the stroke recovery continuum. While the initial months post-stroke hold paramount importance in rehabilitation, evaluating the impact during later stages of neuro-recovery and predicting when a plateau in UE functions occurs will also be crucial.

In future iterations of the network, we will investigate how multimodal integration of this model with other data sources (e.g., kinematics and electromyographic data^75,76^) will improve model performances and robustness.

### F. Conclusion

In this study, we successfully developed and validated a machine-learning model capable of accurately predicting UE motor recovery following a stroke using resting-state EEG recordings obtained in the acute and subacute phases. The proposed approach not only opens up avenues for future research on stroke biomarkers but also provides a data-driven framework to assess the predictive performance of these markers. With additional experiments and validations on larger datasets, our model will constitute the basis for forecasting recovery potentials and customizing rehabilitation strategies, enhancing the ability to tailor interventions to their specific needs from the first hours after the stroke.

## IV. METHODS

### A. Participants and study procedures

Twenty-three acute ischemic stroke survivors were recruited for this study (AOUP-Acute dataset). Inclusion criteria were 18–80 years of age; unilateral motor deficit (with or without other stroke-related symptoms or signs); radiological evidence of unilateral, supratentorial cerebral ischemic lesion (or lesions) in the same arterial territory; stroke occurred in the last 4 days; absent or slight disability before stroke estimated by a modified Rankin Scale of 0–2. Participants were excluded if they were affected by a history of severe cognitive impairment; psychiatric comorbidities; end-stage organic diseases like cardiopulmonary, hepatic, or renal failure, neoplasms; and whatever condition that could strongly reduce life expectancy. Data collection was conducted at the Neurorehabilitation Unit of the University Hospital of Pisa (Italy).

Data collection was conducted at two time points: T0—within the acute post-stroke stage; and T1—during the subacute and early-chronic stages. Between the two assessments, patients followed standard rehabilitative protocols as instructed by their caregivers. During both time points, the NIH Stroke Scale (NIHSS),^77^ and the FMAUE^10^ were administered. The resting-state EEG was recorded for 10 min using a 64-channel analog system at a sampling rate of 256 Hz (“Micromed SD MRI” acquisition System Plus and amplificatory DC-coupled). During the EEG recordings, participants were asked to sit comfortably with their eyes closed, in a relaxed mind-wandering state. Impedances of the recording channels were continuously monitored and readjusted whenever they crossed 10 kΩ. A summary of the demographic and clinical information for the AOUP-Acute dataset is reported in Table I.

**TABLE I.** Demographic and clinical characteristics of the collected sample of the AOUP-Acute dataset. AH: affected hemisphere; FMAUE: Fugl-Meyer Assessment of the Upper Extremity.

ID	Sex	Age at onset (years)	AH	Days since stroke (T0)	Days since stroke (T1)	FMAUE_*T*0_	FMAUE_*T*1_
2	M	53	R	4	105	0	23
3	F	71	L	3	156	66	66
4	M	77	L	3	150	57	62
5	M	58	R	2	86	21	64
6	F	79	L	2	93	66	66
7	F	75	L	3	88	4	43
11	M	73	L	3	250	55	60
12	F	78	L	2	212	0	63
14	M	50	R	4	102	4	12
15	M	50	L	2	303	59	66
16	F	72	R	2	301	64	66
17	M	52	R	2	138	52	60
18	M	64	R	3	175	2	22
20	M	79	L	2	130	66	66
22	M	82	L	1	134	12	59
24	F	53	R	4	96	31	62
25	M	52	R	3	97	6	44
26	M	64	L	3	100	0	19
27	M	64	R	2	88	24	28
30	M	64	L	8	102	32	65
32	M	53	L	3	93	66	66
37	M	60	R	1	108	57	64
38	M	69	L	3	97	8	13

Another dataset was collected from 17 subacute stroke patients at the IRCCS Don Gnocchi Hospital, Florence (FDG-Subacute dataset). Subjects were recruited within 4 weeks from the stroke event, and the follow-up was recorded approximately 3 months after the baseline. The same inclusion criteria and EEG recording protocol were applied as for the AOUP-Acute dataset. The 64-channel EBNeuro Galileo EEG system was used in this case, with a sampling rate of 512 Hz (downsampled to 256 Hz to allow comparison with the AOUP-Acute dataset). Summary information for this dataset is reported in Table II.

**TABLE II.** Demographic and clinical characteristics of the collected sample of the FDG-Subacute dataset. AH: affected hemisphere; FMAUE: Fugl-Meyer Assessment of the Upper Extremity.

ID	Sex	Age at onset (years)	AH	Days since stroke (T0)	Days since stroke (T1)	FMAUE_*T*0_	FMAUE_*T*1_
1	F	64	L	13	95	8	30
2	M	59	L	15	68	61	65
3	M	78	L	15	109	55	64
9	M	51	L	26	89	4	11
10	M	58	L	22	107	65	66
12	F	76	R	28	112	60	60
13	M	78	L	26	97	63	63
15	M	80	L	19	96	6	16
16	M	77	L	19	107	49	60
17	F	70	R	22	97	62	65
19	M	60	R	33	85	4	4
20	M	72	R	45	82	4	6
24	M	52	R	32	96	4	4
27	M	51	R	21	93	66	66
28	M	81	R	24	66	4	9
29	M	76	L	34	82	66	66
30	M	79	L	31	99	63	66

This study was authorized by the local Ethics Committee (*Comitato Etico Regionale per la Sperimentazione Clinica della Regione Toscana, Sezione Area Vasta Nord Ovest*, reference n. 17992), and all participants signed an informed consent according to the requirements of the Declaration of Helsinki.

### B. Proportional recovery rule

We investigated whether recovery patterns from the two datasets followed the model of proportional recovery (PRR),^11^ where

$$\Delta \text{FMAUE}(\text{prop})=\beta *(66-\text{FMAU}E_{T0})+C.$$ (1)

With β = 0.7 and C = 0.4. According to this model, patients are expected to achieve approximately 70% of the difference between their initial FMAUE score and the maximum achievable score.^12^

Based on the PRR, we defined recoverers (REC) as patients having an error in the PRR model lower than 20 FMAUE points and non-recoverers (NO-REC) as the other participants.

### C. EEG preprocessing

A detailed explanation of the preprocessing for removing the physiological and non-physiological artifacts from the raw EEG signals is described in a previous work from our group.^78^ The preprocessing pipeline was developed in Matlab using the EEGLAB toolbox,^79^ and it was composed of two main steps.

First, we obtained robust averaged-referenced signals using the PREP pipeline,^80^ which is composed of (1) PREP high-pass filtering of all channels, using an FIR filter with Hamming window and cutoff frequency = 1 Hz; (2) removal of line noise at 50 Hz and its harmonics; and (3) removal of channels with abnormal and/or uncorrelated activity compared to others. The activities of the remaining channels were then used to estimate a robust average reference, based on the median and interquartile range. Removed channels were interpolated using spherical interpolation, and the remaining artifacts were manually discarded after visual inspection.

Afterward, on the re-referenced and filtered signals, we conducted an independent component analysis (ICA) using the Infomax ICA algorithm,^81^ and we discriminated the brain components from the artifactual ones using a semi-automated procedure. This procedure was composed of two steps: (1) a neural network trained on crowd-sourced data (ICLabel)^82^ provided the probability that each component belonged to one of the following classes: brain, line noise, muscle, eye, channel noise, heart, and other; (2) DIPFIT^83^ was used to perform a single dipole fitting of the independent component map onto a template brain (MNI-152 atlas),^84^ and ICs labeled as “brain” by the neural network with a confidence higher than 75% and having a fitted dipole residual variance below 20% were retained.^83^ All the remaining components were visually inspected and marked either as “brain” or “non-brain” depending on their power spectra and temporal profiles. The reduced IC space was used to reconstruct the channel-level signals. A final visual inspection was performed on these signals to remove remaining artifacts that were not removed by the above ICA procedure.

To account for differences in the lesioned hemisphere, we swapped the left and right channels in the case of a left-hemisphere lesion so that the right-hand side always referred to the lesioned hemisphere.

### D. Feature extraction

A summary of the feature extraction process and subsequent machine-learning model is represented in Fig. 1.

For each participant, we analyzed the central 180 s of the artifact-free EEG signals collected at T0. Feature extraction was conducted on epochs of 10 s duration, testing four values of overlap between consecutive windows: no overlap, 25%, 50%, and 75% of the epoch duration. From each epoch, we extracted several spectral and connectivity features, previously described in the literature as plausible predictors of stroke recovery.

#### 1. Spectral and brain symmetry features

First, we computed an estimate of the power spectral density (PSD) of the signal in each of the recorded channels. For each epoch, we applied the Welch's method (averaged periodogram)^85^ on 2 s continuous windows of EEG signals, using Hamming windows with no overlap. The spectrum was divided into five canonical EEG frequency bands, namely, delta (1–4 Hz), theta (4–8 Hz), alpha (8–13 Hz), beta (13–30 Hz), and gamma (30–48 Hz). For each band, the absolute power was computed with the trapezoid integration method and normalized with respect to the total power between 1 and 48 Hz. The bands' upper bound was set to 48 Hz to reduce the possible influence of residual power line artifacts. The average power was computed as the mean power across all EEG channels. Moreover, the scalp was divided into nine regions of interest (ROIs):^78^ frontal right (FR—channels: Fp2, AF4, AF8, F2, F4, F6, and F8); frontal left (FL—channels Fp1, AF3, AF7, F1, F3, F5, and F7); central right (CR—channels FC2, FC4, FC6, FT8, C2, C4, C6, T4, CP2, CP4, and CP6); central left (CL—channels FC1, FC3, FC5, FT7, C1, C3, C5, T3, CP1, CP3, and CP5); occipital right (OR—channels P2, P4, P6, T6, PO8, PO4, and O2); occipital left (OL—channels P1, P3, P5, T5, PO7, PO3, and O1); frontal (F), as the union of FR with FL; central (C), as the union of CR with CL; and occipital (O), as the union of OR with OL.

Afterward, we extracted the following features (a total of 141):

• Relative power in the five frequency bands on the average spectrum (5 features), within each ROI (45 features), and on the affected and unaffected hemisphere spectra (10 features).
• Delta/alpha ratio (DAR), computed as the ratio between delta and alpha normalized power.^24^ The DAR was computed on the average spectrum (one feature), within each ROI (nine features), and in the affected and unaffected hemisphere spectra (two features).
• (Delta + theta)/(alpha + beta) ratio (DTABR), computed as the ratio between the sum of delta and theta activities and the sum of alpha and beta activities.^24^ The DTABR was computed on the average spectrum (one feature), within each ROI (six features), and in the affected and unaffected hemisphere spectra (two features).
• Pairwise-derived brain symmetry index (pdBSI), calculated as^86,87^
$$\text{pdBSI}=\frac{1}{K}\sum_{n=1}^{K}|\frac{L_{n}-R_{n}}{L_{n}+R_{n}}|.$$ (2)
  In Eq. (2), K is the total number of bins in the band of interest, $L_{n}$ and $R_{n}$ are the sum of PSD in the nth frequency bins. The pdBSI was computed in six frequency bands (i.e., the whole spectrum, delta, theta, alpha, beta, and gamma bands) and four ROIs (i.e., F, C, O, and on average), for a total of 24 features.
• Directional pairwise-derived brain symmetry index (dirpdBSI), calculated as^26^
$$\text{dirpdBSI}=\frac{1}{K}\sum_{n=1}^{K}\frac{L(n)-R(n)}{L(n)+R(n)}.$$ (3)
  The terms in Eq. (3) are the same as for Eq. (2), but the absolute value is removed to capture the directionality of the relation. Again, the dirpdBSI was computed in six frequency bands (i.e., the whole spectrum, delta, theta, alpha, beta, and gamma bands) and four ROIs (i.e., F, C, O, and on average), for a total of 24 features.
• Individual alpha frequency (IAF),^88^ computed as the center of gravity frequency of the power spectrum in the alpha band
$$\text{IAF}=\frac{1}{P_{n}}\sum_{n=1}^{K}P_{n}\cdot f_{n}.$$ (4)
  In Eq. (4), K is the total number of bins belonging to the alpha band, $P_{n}$ is the PSD value in the nth bin, and $f_{n}$ is the frequency at the center of the nth bin. The IAF was computed on the average spectrum, within each ROI, and in the affected and unaffected hemisphere spectra, for a total of 12 features.

EEG preprocessing and spectral analysis were performed using Matlab with the EEGLAB toolbox.^79^

#### 2. Connectivity and network features

Connectivity among the ROIs described above was computed by estimating the imaginary component of the coherency spectrum^89^ in the five frequency bands, defined as

$$Coh_{ij}(f)=ℑ(\frac{S_{ij}(f)}{\sqrt{S_{ii}(f)S_{jj}(f)}}).$$ (5)

In Eq. (5), $S_{ij}(f)$ is the cross-spectrum between ROIs i and j at frequency f, while $S_{ii}(f)$ and $S_{jj}(f)$ are the spectra of ROIs i and j at frequency f. ℑ indicates the imaginary part of the measure. The imaginary component of the coherency is used instead of the coherence measure to disentangle the effects of zero-lag connectivity, mostly associated with volume conduction effects.^89^ From the full connectivity matrix, we obtained a sparse network representation by pruning the weights with a proportional threshold, set as the highest percentile that kept all components connected (1% percentile increase).^78^ From the sparse graph representation, the following connectivity and network features were extracted for each band (for a total of 80 features):

• Average degree of connections, computed as the mean number of connections retained after pruning (five features).
• Average strength of connections, computed as the mean weighted node degree of connections,^60^ namely the average sum of all coherence (five features).
• Weighted characteristic path length,^90,91^ indicating the average functional distance between two ROIs (five features).
• Average weighted clustering coefficient:^90,91^ a measure that represents the tendency of the network to form clusters of nodes (closed graphs) (five features).
• Small-world omega index.^78,92^ The small-world property indicates the propensity of the network to be tightly interconnected (low characteristic path length) while being highly clustered (high clustering coefficient) (five features). The omega measure compares $L_{w}$ and $C_{w}$ with the characteristic path length of an equivalent random network ( $L_{r}$) and the clustering coefficient of an equivalent lattice network ( $C_{l}$), respectively:
$$\omega =\frac{L_{r}}{L_{w}}-\frac{C_{w}}{C_{l}}.$$ (6)
• Rich Club coefficient, computed as the average number of edges that connect to nodes having strength higher than the 75th percentile of the coherency distribution, divided by the total strength of the node (five features).
• Degree and strength of connections of affected and unaffected motor ROIs^63^ (20 features).
• Path length and clustering coefficients of the affected and unaffected motor ROIs (20 features).
• Local efficiency of the affected and unaffected motor ROIs,^93^ measured as the inverse of the short path between ROI pairs (ten features).

Connectivity estimation was performed using the Fieldtrip toolbox,^94^ while network metrics were computed using the Brain Connectivity Toolbox.^95^

The $\text{FMAU}E_{T0}$ was included in the feature set, along with the number of days from stroke onset and time-to-follow-up examination. Thus, for each participant and each EEG epoch, we obtained 224 features.

### E. Regression analysis

The goal of the regression analysis was to build and validate an algorithm to predict the $\text{FMAU}E_{T1}$ given the predictors extracted at T0. To achieve this goal, we implemented a feed-forward, fully connected neural network for regression (*StrokeRecovNet*) that, given a small set of EEG features extracted from the EEG recordings, estimates the FMAUE at the follow-up. Previous research primarily focused on defining EEG descriptors to predict motor impairment and to develop traditional machine-learning models based on these descriptors. Our work emphasizes the development of a more adaptable model to accommodate a wide array of candidate features while avoiding the complexities of extensive parameter training requirements typical of deep learning models. *StrokeRecovNet* efficiently utilizes the full information content of the input features. Crucially, it achieves this without relying on large datasets typically necessary for training more complex models.

To train, validate, and test the performance of the model, an LOSO approach was adopted. Patients with a maximum baseline FMA-UE score (i.e., 66) were excluded from the testing process but retained for training. In the FDG-Subacute cohort we also excluded from the testing those who reached the ceiling at follow-up to avoid distorted recovery estimates.

*StrokeRecovNet* was assessed in three different experiments:

1. Experiment #1: Training, validation, and testing with nested LOSO-CV within the AOUP-Acute dataset.
2. Experiment #2: Training, validation, and testing with nested LOSO-CV on the FDG-Subacute dataset only.
3. Experiment #3: Training, validation, and testing with nested LOSO-CV on the combination of the AOUP-Acute and FDG-Subacute datasets (i.e., FDG + AOUP-Combined datasets), with performance evaluation on the AOUP dataset.

The rationale for these experiments was to test whether acute data alone were sufficient to accurately predict FMAUE at follow-up (experiment #1), whether subacute data were predictable with the same set of EEG features (experiment #2), and whether training on a larger dataset of acute and subacute patients could improve the performance of the model (experiment #3). Although acute and subacute EEG reflect different stages of neuroplasticity, we used the combined dataset solely to test whether more stable subacute patterns could support prediction in the physiologically more variable acute phase. Importantly, testing was always performed only on acute patients, so the experiment does not assume phase equivalence but assesses whether subacute information provides useful predictive structure.

All features in the training set were ranked using either the ReliefF^96^ or mRMR^97^ feature selection algorithm, depending on which achieved the best performance. These methods are effective because ReliefF captures feature relevance by considering local instance neighborhoods, while mRMR balances maximizing relevance and minimizing redundancy among features, hence effectively reducing feature collinearity issues. We fine-tuned the regression algorithm by selecting the top four features and testing the best combination among all of them. To balance computational feasibility with model stability, we restricted the input space to the top four features identified through the ReliefF and mRMR selection schemes. This approach was chosen because the number of grid-search iterations grows non-linearly with feature count, particularly when paired with Leave-One-Subject-Out cross-validation, which requires retraining the model for each participant. Limiting the feature set therefore ensured tractable optimization while preserving interpretability and reducing redundancy among highly correlated EEG predictors.

We added as fixed inputs to the model the $\text{FMAU}E_{T0}$, the time since stroke (days), and the time of prediction (days), as to better facilitate the blending of the two datasets. This allowed us to work with a focused set of features, conducting an extensive search for the best feature combinations during the LOSO cross-validation, using the root mean square error (RMSE) as a performance metric. The testing performance was compared to the PRR in terms of median absolute error (MAE).

### F. Cross-validation procedure

During the LOSO-CV, the regression models were trained to predict the $\text{FMAU}E_{T1}$ of the validation epochs (i.e., epochs belonging to the participant left out at each iteration of the LOSO). Then, the median of the predicted values of all validation epochs was used as the $\text{FMAU}E_{T1}$ of the left-out participant. To find the best model, for each algorithm, we conducted an exhaustive search among the following hyperparameters:

• Overlap between consecutive EEG epochs: no overlap, 25%, 50%, and 75% of the epoch duration.
• Input features: the regression algorithm was fed with all possible combinations of the best-performing EEG features (based on the feature selection ranking), ranging from one to four selected features (we always included $\text{FMAU}E_{T0}$, days since stroke and days to T1 as additional features of all models).
• Feature selection method: ReliefF or mRMR.
• *StrokeRecovNet* layer configuration: we tested six possible network configurations, with one, two, or three hidden layers, respectively, with the following neurons-per-layer configurations (8; 16; 32; [16, 8]; [32, 16]; [32, 16, 8]).
• Batch size (64, 128, full-batch).

Each neural network model evaluated during training was trained for 20 epochs using the Adam optimizer, with a set learning rate of 0.01. ReLU activation functions were selected.

The RMSE between the predicted and true FMAUE_*T*1_ was used to assess the model's performance in the validation loop. The combination of the model's hyperparameters, input features, and window overlap that minimized the RMSE on the validation set was used to determine the best regression model.

### G. Feature consistency scores

We investigated the degree of overlap between the features selected in each experiment to verify whether model representation changed across stroke phases. As we extracted a large number of possibly related features, we did not compare directly whether the same feature set was selected across models. We computed two measures of more general feature consistency across models: the domain consistency score and the cluster consistency score.

To evaluate domain consistency, we grouped features into functional domains (i.e., spectral metrics, brain symmetry, node connectivity, and network metrics). Each subject's selected features were mapped to these domains, and the selection frequency of each domain was computed as the proportion of subjects selecting at least one feature within each domain. Pairwise domain consistency between datasets was then assessed using a weighted similarity score. First, we computed the mean domain selection frequency across all datasets to derive domain-specific weights. For each dataset pair, we calculated the weighted absolute differences in selection frequencies for each domain and normalized this value to obtain a similarity score bounded between 0 and 1. An overall domain consistency score was also computed as the average of all pairwise similarity scores, providing a global measure of domain-level agreement across datasets.

The cluster consistency score was instead computed to account for the possibility that predefined domains would not completely capture interrelations between features (e.g., correlations within metrics extracted from the same frequency band). To compute cluster consistency, we first derived feature clusters by applying hierarchical agglomerative clustering with complete linkage to the absolute Spearman correlation matrix of all features in the dataset. The resulting clusters were defined by a threshold on the correlation-derived distance metric (1 − correlation), with a cutoff of 0.7 (i.e., correlation ≥0.3).

Next, for each dataset, we computed the relative frequency with which each cluster was selected among the best feature sets identified per subject. Specifically, for each cluster, we recorded the proportion of subjects whose selected features included at least one feature belonging to that cluster. This yielded a vector of cluster selection frequencies per dataset. Cluster consistency between two datasets was then quantified using the Jaccard similarity between the sets of clusters that had a non-zero selection frequency in each dataset.

### H. Statistical analyses

The performance of the models trained in each experiment was compared to that of PRR by means of a bootstrap permutation test (n = 10 000).

We investigated differences between recoverers' and non-recoverers' features by performing a Mann–Whitney U-test to compare the feature values between recoverers and non-recoverers for each feature that was selected at least once in the final model (we applied Bonferroni correction by the total tested number of features; for visualization purposes, we only display the violin plots of the top four selected significant features). Due to sample size constraints, we compared feature values at the window level (pooling together all the windows from all subjects within a group), rather than at the subject level. To this purpose, we investigated features extracted from the no overlap dataset.

## SUPPLEMENTARY MATERIAL

See the supplementary material for the best hyperparameter frequency for each of the trained networks (Table S1), the lesion location information for patients from both datasets (Table S2), the full results of model training (Table S3), the results of model training on a fixed feature set (Table S4), the statistical results of the comparison of model errors with possible confounding factors (Tables S5 and S6), and the results of cross-dataset predictions (Table S7).

## Data Availability

The data that support the findings of this study are available from the corresponding author upon reasonable request.

## References

[1] GBD 2019 Stroke Collaborators, Lancet Neurol. 20, 795–820 (2021). 10.1016/S1474-4422(21)00252-0 34487721 PMC8443449

[2] E. R. Coleman, R. Moudgal, K. Lang, H. I. Hyacinth, O. O. Awosika, B. M. Kissela, and W. Feng, Curr. Atheroscler. Rep. 19, 59 (2017). 10.1007/s11883-017-0686-6 29116473 PMC5802378

[3] A. S. Go, D. Mozaffarian, V. L. Roger, E. J. Benjamin, J. D. Berry, M. J. Blaha, S. Dai, E. S. Ford, C. S. Fox, S. Franco, H. J. Fullerton, C. Gillespie, S. M. Hailpern, J. A. Heit, V. J. Howard, M. D. Huffman, S. E. Judd, B. M. Kissela, S. J. Kittner, D. T. Lackland, J. H. Lichtman, L. D. Lisabeth, R. H. Mackey, D. J. Magid, G. M. Marcus, A. Marelli, D. B. Matchar, D. K. McGuire, E. R. Mohler, C. S. Moy, M. E. Mussolino, R. W. Neumar, G. Nichol, D. K. Pandey, N. P. Paynter, M. J. Reeves, P. D. Sorlie, J. Stein, A. Towfighi, T. N. Turan, S. S. Virani, N. D. Wong, D. Woo, M. B. Turner, and American Heart Association Statistics Committee and Stroke Statistics Subcommittee, Circulation 129, e28 (2014). 10.1161/01.cir.0000441139.02102.80 24352519 PMC5408159

[4] S. M. Hatem, G. Saussez, M. della Faille, V. Prist, X. Zhang, D. Dispa, and Y. Bleyenheuft, Front. Hum. Neurosci. 10, 442 (2016). 10.3389/fnhum.2016.00442 27679565 PMC5020059

[5] K. S. Hayward, S. F. Kramer, V. Thijs, J. Ratcliffe, N. S. Ward, L. Churilov, L. Jolliffe, D. Corbett, G. Cloud, T. Kaffenberger, A. Brodtmann, J. Bernhardt, and N. A. Lannin, Syst. Rev. 8, 187 (2019). 10.1186/s13643-019-1093-6 31345263 PMC6657039

[6] R. M. Banjai, S. M. S. F. de Freitas, F. P. da Silva, and S. R. Alouche, Top. Stroke Rehabil. 25, 174 (2018). 10.1080/10749357.2017.1406177 29226780

[7] P. Raghavan, Phys. Med. Rehabil. Clin. North Am. 26, 599 (2015). 10.1016/j.pmr.2015.06.008 PMC4844548 26522900

[8] D. S. Nichols-Larsen, P. C. Clark, A. Zeringue, A. Greenspan, and S. Blanton, Stroke 36, 1480 (2005). 10.1161/01.STR.0000170706.13595.4f 15947263

[9] C. Stinear, Lancet Neurol. 9, 1228 (2010). 10.1016/S1474-4422(10)70247-7 21035399

[10] A. R. Fugl-Meyer, L. Jääskö, I. Leyman, S. Olsson, and S. Steglind, Scand. J. Rehabil. Med. 7, 13 (1975). 10.2340/1650197771331 1135616

[11] C. Winters, E. E. H. van Wegen, A. Daffertshofer, and G. Kwakkel, Neurorehabil. Neural Repair 29, 614 (2015). 10.1177/1545968314562115 25505223

[12] S. Prabhakaran, E. Zarahn, C. Riley, A. Speizer, J. Y. Chong, R. M. Lazar, R. S. Marshall, and J. W. Krakauer, Neurorehabil. Neural Repair 22, 64 (2008). 10.1177/1545968307305302 17687024

[13] R. Kundert, J. Goldsmith, J. M. Veerbeek, J. W. Krakauer, and A. R. Luft, Neurorehabil. Neural Repair 33, 876 (2019). 10.1177/1545968319872996 31524062 PMC6854610

[14] R. van der Vliet, R. W. Selles, E.-R. Andrinopoulou, R. Nijland, G. M. Ribbers, M. A. Frens, C. Meskers, and G. Kwakkel, Ann. Neurol. 87, 383 (2020). 10.1002/ana.25679 31925838 PMC7065018

[15] T. M. H. Hope, K. Friston, C. J. Price, A. P. Leff, P. Rotshtein, and H. Bowman, Brain 142, 15 (2019). 10.1093/brain/awy302 30535098 PMC6308308

[16] J. W. Krakauer and R. S. Marshall, Ann. Neurol. 78, 845 (2015). 10.1002/ana.24537 26435166

[17] A. K. Bonkhoff, T. Hope, D. Bzdok, A. G. Guggisberg, R. L. Hawe, S. P. Dukelow, A. K. Rehme, G. R. Fink, C. Grefkes, and H. Bowman, Brain 143, 2189 (2020). 10.1093/brain/awaa146 32601678

[18] A. K. Bonkhoff, T. Hope, D. Bzdok, A. G. Guggisberg, R. L. Hawe, S. P. Dukelow, F. Chollet, D. J. Lin, C. Grefkes, and H. Bowman, J. Neurol., Neurosurg. Psychiatry 93, 369 (2022). 10.1136/jnnp-2021-327211 34937750

[19] P. J. Hand, J. M. Wardlaw, A. M. Rowat, J. A. Haisma, R. I. Lindley, and M. S. Dennis, J. Neurol., Neurosurg. Psychiatry 76, 1525 (2005). 10.1136/jnnp.2005.062539 16227544 PMC1739389

[20] P. J. Koch, C.-H. Park, G. Girard, E. Beanato, P. Egger, G. G. Evangelista, J. Lee, M. J. Wessel, T. Morishita, G. Koch, J.-P. Thiran, A. G. Guggisberg, C. Rosso, Y.-H. Kim, and F. C. Hummel, Brain 144, 2107 (2021). 10.1093/brain/awab082 34237143 PMC8370413

[21] A. G. Guggisberg, P. Nicolo, L. G. Cohen, A. Schnider, and E. R. Buch, Neurorehabil. Neural Repair 31, 1029 (2017). 10.1177/1545968317740634 29130824 PMC6368856

[22] E. Zarahn, L. Alon, S. L. Ryan, R. M. Lazar, M.-S. Vry, C. Weiller, R. S. Marshall, and J. W. Krakauer, Cereb. Cortex 21, 2712 (2011). 10.1093/cercor/bhr047 21527788 PMC3209795

[23] W. D. Byblow, C. M. Stinear, P. A. Barber, M. A. Petoe, and S. J. Ackerley, Ann. Neurol. 78, 848 (2015). 10.1002/ana.24472 26150318

[24] S. Finnigan, A. Wong, and S. Read, Clin. Neurophysiol. 127, 1452 (2016). 10.1016/j.clinph.2015.07.014 26251106

[25] A. M. Chiarelli, P. Croce, G. Assenza, A. Merla, G. Granata, N. M. Giannantoni, V. Pizzella, F. Tecchio, and F. Zappasodi, Int. J. Neural Syst. 30, 2050067 (2020). 10.1142/S0129065720500677 33236654

[26] M. Saes, S. B. Zandvliet, A. S. Andringa, A. Daffertshofer, J. W. R. Twisk, C. G. M. Meskers, E. E. H. van Wegen, and G. Kwakkel, Neurorehabil. Neural Repair 34, 389 (2020). 10.1177/1545968320905797 32249674

[27] S. Finnigan and M. J. A. M. van Putten, Clin. Neurophysiol. 124, 10 (2013). 10.1016/j.clinph.2012.07.003 22858178

[28] A. A. Vatinno, A. Simpson, V. Ramakrishnan, H. S. Bonilha, L. Bonilha, and N. J. Seo, Neurorehabil. Neural Repair 36, 255 (2022). 10.1177/15459683221078294 35311412 PMC9007868

[29] L. Sutcliffe, H. Lumley, L. Shaw, R. Francis, and C. I. Price, BMC Emerg. Med. 22, 29 (2022). 10.1186/s12873-022-00585-w 35227206 PMC8883639

[30] M. Saes, C. G. M. Meskers, A. Daffertshofer, E. E. H. van Wegen, and G. Kwakkel, Clin. Neurophysiol. 132, 56 (2021). 10.1016/j.clinph.2020.09.031 33248434

[31] R. V. A. Sheorajpanday, G. Nagels, A. J. T. M. Weeren, M. J. A. M. van Putten, and P. P. De Deyn, Clin. Neurophysiol. 122, 874 (2011). 10.1016/j.clinph.2010.07.028 20961806

[32] J. M. Cassidy, J. I. Mark, and S. C. Cramer, Brain 145, 1211 (2022). 10.1093/brain/awab469 34932786 PMC9630718

[33] N. Meneghetti, M. Lassi, V. Massa, S. Micera, A. Mazzoni, C. Alia, and A. Bandini, APL Bioeng. 9, 026108 (2025). 10.1063/5.0263191 40270920 PMC12017806

[34] N. Riahi, V. A. Vakorin, and C. Menon, IEEE Trans. Neural Syst. Rehabil. Eng. 28, 860 (2020). 10.1109/TNSRE.2020.2978381 32149693

[35] R. I. Carino-Escobar, R. Valdés-Cristerna, P. Carrillo-Mora, M. A. Rodriguez-Barragan, C. Hernandez-Arenas, J. Quinzaños-Fresnedo, O. Arias-Carrión, and J. Cantillo-Negrete, J. Neural Eng. 18, 046057 (2021). 10.1088/1741-2552/abfc1e 33906163

[36] X. Zhang, R. D'Arcy, L. Chen, M. Xu, D. Ming, and C. Menon, Sensors 20, 5487 (2020). 10.3390/s20195487 32992698 PMC7582505

[37] M. Lassi, A. Bandini, V. Spina, V. Azzollini, S. Dalise, A. Mazzoni, C. Chisari, and S. Micera, in *2023 11th International IEEE/EMBS Conference on Neural Engineering (NER)* (IEEE, 2023), pp. 1–4.

[38] J. D. Schaechter, Prog. Neurobiol. 73, 61 (2004). 10.1016/j.pneurobio.2004.04.001 15193779

[39] F. Pichiorri, M. Petti, S. Caschera, L. Astolfi, F. Cincotti, and D. Mattia, Eur. J. Neurosci. 47, 158 (2018). 10.1111/ejn.13797 29247485

[40] S. J. Page, G. D. Fulk, and P. Boyne, Phys. Ther. 92, 791 (2012). 10.2522/ptj.20110009 22282773

[41] S. Hiragami, Y. Inoue, and K. Harada, J. Phys. Ther. Sci. 31, 917 (2019). 10.1589/jpts.31.917 31871377 PMC6879402

[42] A. K. Bonkhoff, M. D. Schirmer, M. Bretzner, S. Hong, R. W. Regenhardt, K. L. Donahue, M. J. Nardin, A. V. Dalca, A. Giese, M. R. Etherton, B. L. Hancock, S. J. T. Mocking, E. C. McIntosh, J. Attia, J. W. Cole, A. Donatti, C. J. Griessenauer, L. Heitsch, L. Holmegaard, K. Jood, J. Jimenez‐Conde, S. J. Kittner, R. Lemmens, C. R. Levi, C. W. McDonough, J. F. Meschia, C. Phuah, S. Ropele, J. Rosand, J. Roquer, T. Rundek, R. L. Sacco, R. Schmidt, P. Sharma, A. Slowik, A. Sousa, T. M. Stanne, D. Strbian, T. Tatlisumak, V. Thijs, A. Vagal, J. Wasselius, D. Woo, R. Zand, P. F. McArdle, B. B. Worrall, C. Jern, A. G. Lindgren, J. Maguire, O. Wu, and N. S. Rost, Hum. Brain Mapp. 44, 1579 (2023). 10.1002/hbm.26159 36440953 PMC9921242

[43] J. Wu, R. Srinivasan, E. Burke Quinlan, A. Solodkin, S. L. Small, and S. C. Cramer, J. Neurophysiol. 115, 2399 (2016). 10.1152/jn.00978.2015 26936984 PMC4922461

[44] P. Liuzzi, A. Grippo, A. Sodero, C. Castagnoli, I. Pellegrini, R. Burali, T. Toci, T. Barretta, A. Mannini, B. Hakiki, C. Macchi, F. Lolli, and F. Cecchi, Neurophysiol. Clin. 54, 102952 (2024). 10.1016/j.neucli.2024.102952 38422721

[45] F. Vecchio, C. Tomino, F. Miraglia, F. Iodice, C. Erra, R. Di Iorio, E. Judica, F. Alù, M. Fini, and P. M. Rossini, Int. J. Psychophysiol. 146, 133 (2019). 10.1016/j.ijpsycho.2019.09.012 31648028

[46] J. M. Cassidy, A. Wodeyar, R. Srinivasan, and S. C. Cramer, Hum. Brain Mapp. 42, 5636 (2021). 10.1002/hbm.25643 34435705 PMC8559506

[47] J. M. Cassidy, A. Wodeyar, J. Wu, K. Kaur, A. K. Masuda, R. Srinivasan, and S. C. Cramer, Stroke 51, 1442 (2020). 10.1161/STROKEAHA.120.028932 32299324 PMC7188582

[48] F. Zappasodi, P. Croce, A. Giordani, G. Assenza, N. M. Giannantoni, P. Profice, G. Granata, P. M. Rossini, and F. Tecchio, Brain Topogr. 30, 698 (2017). 10.1007/s10548-017-0572-0 28547185

[49] T. Hoshino, K. Oguchi, K. Inoue, A. Hoshino, and M. Hoshiyama, Top. Stroke Rehabil. 28, 614 (2021). 10.1080/10749357.2020.1864986 33351724

[50] T. Kawano, N. Hattori, Y. Uno, M. Hatakenaka, H. Yagura, H. Fujimoto, T. Yoshioka, M. Nagasako, H. Otomune, K. Kitajo, and I. Miyai, Neurorehabil. Neural Repair 34, 711 (2020). 10.1177/1545968320935820 32691673 PMC7457459

[51] A. Agius Anastasi, O. Falzon, K. Camilleri, M. Vella, and R. Muscat, Stroke Res. Treat. 2017, 8276136. 10.1155/2017/8276136 28251015 PMC5304313

[52] M. Lassi, S. Dalise, A. Bandini, V. Spina, V. Azzollini, M. Vissani, S. Micera, A. Mazzoni, and C. Chisari, Eur. J. Phys. Rehabil. Med. 60, 13 (2024). 10.23736/S1973-9087.23.07922-4 37987741 PMC10932487

[53] M. Ajčević, G. Furlanis, A. Miladinović, A. Buoite Stella, P. Caruso, M. Ukmar, M. A. Cova, M. Naccarato, A. Accardo, and P. Manganotti, Ann. Biomed. Eng. 49, 2150 (2021). 10.1007/s10439-021-02735-w 33604799 PMC8455382

[54] C. Fanciullacci, F. Bertolucci, G. Lamola, A. Panarese, F. Artoni, S. Micera, B. Rossi, and C. Chisari, Front. Hum. Neurosci. 11, 385 (2017). 10.3389/fnhum.2017.00385 28804453 PMC5532374

[55] Y. Sato, O. Schmitt, Z. Ip, G. Rabiller, S. Omodaka, T. Tominaga, A. Yazdan-Shahmorad, and J. Liu, J. Cereb. Blood Flow Metab. 42, 1753 (2022). 10.1177/0271678X221105677 35754347 PMC9536122

[56] F. Taddeini, G. Avvenuti, A. A. Vergani, J. Carpaneto, F. Setti, D. Bergamo, L. Fiorini, P. Pietrini, E. Ricciardi, G. Bernardi, and A. Mazzoni, eNeuro 12, ENEURO.0354-24.2024 (2025). 10.1523/ENEURO.0354-24.2024 PMC11810548 39870524

[57] G. Buzsáki and X.-J. Wang, Annu. Rev. Neurosci. 35, 203 (2012). 10.1146/annurev-neuro-062111-150444 22443509 PMC4049541

[58] M. Hazime, M. Alasoadura, R. Lamtahri, P. Quilichini, J. Leprince, D. Vaudry, and J. Chuquet, Exp. Neurol. 341, 113696 (2021). 10.1016/j.expneurol.2021.113696 33727098

[59] H. E. Rossiter, E. M. Davis, E. V. Clark, M.-H. Boudrias, and N. S. Ward, NeuroImage 91, 360 (2014). 10.1016/j.neuroimage.2014.01.012 24440529 PMC3988925

[60] P. Nicolo, S. Rizk, C. Magnin, M. D. Pietro, A. Schnider, and A. G. Guggisberg, Brain 138, 3048 (2015). 10.1093/brain/awv200 26163304

[61] E. Carrera and G. Tononi, Brain 137, 2408 (2014). 10.1093/brain/awu101 24871646

[62] J. C. Griffis, N. V. Metcalf, M. Corbetta, and G. L. Shulman, Cell Rep. 28, 2527 (2019). 10.1016/j.celrep.2019.07.100 31484066 PMC7032047

[63] S. Dubovik, R. Ptak, T. Aboulafia, C. Magnin, N. Gillabert, L. Allet, J.-M. Pignat, A. Schnider, and A. G. Guggisberg, Behav. Neurol. 26, 187 (2013). 10.1155/2013/109764 22713421 PMC5214220

[64] N. Meneghetti, E. Vannini, and A. Mazzoni, J. Physiol. 602, 1017 (2024). 10.1113/JP283858 38372352

[65] A. Palmisano, S. Pandit, C. L. Smeralda, I. Demchenko, S. Rossi, L. Battelli, D. Rivolta, V. Bhat, and E. Santarnecchi, Life 14(5), 578 (2024). 10.3390/life14050578 38792599 PMC11122172

[66] A. Antonioni, M. Galluccio, R. Toselli, A. Baroni, G. Fregna, N. Schincaglia, G. Milani, M. Cosma, G. Ferraresi, M. Morelli, I. Casetta, A. De Vito, S. Masiero, N. Basaglia, P. Malerba, G. Severini, and S. Straudi, Clin. EEG Neurosci. 55, 465 (2024). 10.1177/15500594231209397 37859431

[67] R. M. Maura, S. Rueda Parra, R. E. Stevens, D. L. Weeks, E. T. Wolbrecht, and J. C. Perry, J. Neuroeng. Rehabil. 20, 21 (2023). 10.1186/s12984-023-01142-7 36793077 PMC9930366

[68] A. Antonioni, M. Galluccio, A. Baroni, G. Fregna, T. Pozzo, G. Koch, F. Manfredini, L. Fadiga, P. Malerba, and S. Straudi, Ann. Phys. Rehabil. Med. 67, 101817 (2024). 10.1016/j.rehab.2024.101817 38479116

[69] S. Boni, M. Galluccio, A. Baroni, C. Martinuzzi, G. Milani, M. Emanuele, S. Straudi, L. Fadiga, and T. Pozzo, J. Clin. Med. 12, 1327 (2023). 10.3390/jcm12041327 36835865 PMC9961867

[70] H. Choi, H. Lim, J. W. Kim, Y. J. Kang, J. Ku, H. Choi, H. Lim, J. W. Kim, Y. J. Kang, and J. Ku, Electronics 8, 1466 (2019). 10.3390/electronics8121466

[71] U. N. Ismail, N. Yahya, and H. A. Manan, Brain Res. 1840, 149023 (2024). 10.1016/j.brainres.2024.149023 38815644

[72] G. Milani, A. Antonioni, A. Baroni, P. Malerba, and S. Straudi, Brain Topogr. 35, 651 (2022). 10.1007/s10548-022-00915-y 36136166 PMC9684227

[73] S. Campagnini, C. Arienti, M. Patrini, P. Liuzzi, A. Mannini, and M. C. Carrozza, J. Neuroeng. Rehabil. 19, 54 (2022). 10.1186/s12984-022-01032-4 35659246 PMC9166382

[74] S. M. Lundberg and S.-I. Lee, in *Proceedings of the 31st International Conference on Neural Information Processing Systems, NIPS'17* (Curran Associates Inc., Red Hook, NY, 2017), pp. 4768–4777.

[75] L. Privitera, M. Lassi, S. Dalise, V. Azzollini, L. Maggiani, A. Guggisberg, A. Mazzoni, C. Chisari, S. Micera, and A. Bandini, “A multimodal machine learning approach to forecast upper limb motor recovery after stroke using kinematic and electromyographic data,” Research Square (2025). 10.21203/rs.3.rs-6629302/v1 PMC12825236 41486155

[76] L. Privitera, M. Lassi, S. Dalise, V. Azzolini, F. Vercillo, L. Maggiani, A. Mazzoni, C. Chisari, S. Micera, and A. Bandini, in *2024 10th IEEE RAS/EMBS International Conference for Biomedical Robotics and Biomechatronics (BioRob)* (IEEE, 2024), pp. 907–912 ISSN: 2155-1782.

[77] S. E. Kasner, J. A. Chalela, J. M. Luciano, B. L. Cucchiara, E. C. Raps, M. L. McGarvey, M. B. Conroy, and A. R. Localio, Stroke 30, 1534 (1999). 10.1161/01.STR.30.8.1534 10436096

[78] M. Lassi, C. Fabbiani, S. Mazzeo, R. Burali, A. A. Vergani, G. Giacomucci, V. Moschini, C. Morinelli, F. Emiliani, M. Scarpino, S. Bagnoli, A. Ingannato, B. Nacmias, S. Padiglioni, S. Micera, S. Sorbi, A. Grippo, V. Bessi, and A. Mazzoni, NeuroImage 38, 103407 (2023). 10.1016/j.nicl.2023.103407 37094437 PMC10149415

[79] A. Delorme and S. Makeig, J. Neurosci. Methods 134, 9 (2004). 10.1016/j.jneumeth.2003.10.009 15102499

[80] N. Bigdely-Shamlo, T. Mullen, C. Kothe, K.-M. Su, and K. A. Robbins, Front. Neuroinf. 9, 16 (2015). 10.3389/fninf.2015.00016 PMC4471356 26150785

[81] D. Langlois, S. Chartier, and D. Gosselin, Tutorials Quant. Methods Psychol. 6(1), 31–38 (2010). 10.20982/tqmp.06.1.p031

[82] L. Pion-Tonachini, K. Kreutz-Delgado, and S. Makeig, NeuroImage 198, 181 (2019). 10.1016/j.neuroimage.2019.05.026 31103785 PMC6592775

[83] A. Delorme, J. Palmer, J. Onton, R. Oostenveld, and S. Makeig, PLoS One 7, e30135 (2012). 10.1371/journal.pone.0030135 22355308 PMC3280242

[84] A. C. Evans, A. L. Janke, D. L. Collins, and S. Baillet, NeuroImage 62, 911 (2012). 10.1016/j.neuroimage.2012.01.024 22248580

[85] P. Welch, IEEE Trans. Audio Electroacoust. 15, 70 (1967). 10.1109/TAU.1967.1161901

[86] M. J. van Putten and D. L. Tavy, Stroke 35, 2489 (2004). 10.1161/01.STR.0000144649.49861.1d 15472102

[87] R. V. A. Sheorajpanday, G. Nagels, A. J. T. M. Weeren, M. J. A. M. van Putten, and P. P. De Deyn, Clin. Neurophysiol. 120, 845 (2009). 10.1016/j.clinph.2009.02.171 19375386

[88] A. W. Corcoran, P. M. Alday, M. Schlesewsky, and I. Bornkessel-Schlesewsky, Psychophysiology 55, e13064 (2018). 10.1111/psyp.13064 29357113

[89] G. Nolte, O. Bai, L. Wheaton, Z. Mari, S. Vorbach, and M. Hallett, Clin. Neurophysiol. 115, 2292 (2004). 10.1016/j.clinph.2004.04.029 15351371

[90] P. Caliandro, F. Vecchio, F. Miraglia, G. Reale, G. Della Marca, G. La Torre, G. Lacidogna, C. Iacovelli, L. Padua, P. Bramanti, and P. M. Rossini, Neurorehabil. Neural Repair 31, 81 (2017). 10.1177/1545968316662525 27511048

[91] C. Fanciullacci, A. Panarese, V. Spina, M. Lassi, A. Mazzoni, F. Artoni, S. Micera, and C. Chisari, Front. Hum. Neurosci. 15, 669915 (2021). 10.3389/fnhum.2021.669915 34276326 PMC8281978

[92] D. J. Watts and S. H. Strogatz, Nature 393, 440 (1998). 10.1038/30918 9623998

[93] F. de Vico Fallani, L. Astolfi, F. Cincotti, D. Mattia, D. la Rocca, E. Maksuti, S. Salinari, F. Babiloni, B. Vegso, G. Kozmann, and Z. Nagy, Anat. Rec. 292, 2023–2031 (2009). 10.1002/ar.20965 19943355

[94] R. Oostenveld, P. Fries, E. Maris, and J.-M. Schoffelen, Comput. Intell. Neurosci. 2011, 156869. 10.1155/2011/156869 21253357 PMC3021840

[95] M. Rubinov and O. Sporns, NeuroImage 52, 1059 (2010). 10.1016/j.neuroimage.2009.10.003 19819337

[96] M. Robnik-Šikonja and I. Kononenko, Mach. Learn. 53, 23 (2003). 10.1023/A:1025667309714

[97] M. Radovic, M. Ghalwash, N. Filipovic, and Z. Obradovic, BMC Bioinf. 18, 9 (2017). 10.1186/s12859-016-1423-9 PMC5209828 28049413

